# Differential Diagnosis of Inflammatory Arthropathies by Musculoskeletal Ultrasonography: A Systematic Literature Review

**DOI:** 10.3389/fmed.2020.00141

**Published:** 2020-05-07

**Authors:** Garifallia Sakellariou, Carlo Alberto Scirè, Antonella Adinolfi, Alberto Batticciotto, Alessandra Bortoluzzi, Andrea Delle Sedie, Orazio De Lucia, Christian Dejaco, Oscar Massimiliano Epis, Emilio Filippucci, Luca Idolazzi, Andrea Picchianti Diamanti, Alen Zabotti, Annamaria Iagnocco, Georgios Filippou

**Affiliations:** ^1^Division of Rheumatology, IRCCS Policlinico San Matteo Foundation, University of Pavia, Pavia, Italy; ^2^UOC e Sezione di Reumatologia - Dipartimento di Scienze Mediche, Università degli Studi di Ferrara, Ferrara, Italy; ^3^Società Italiana di Reumatologia, Unità Epidemiologica, Milan, Italy; ^4^Rheumatology Unit, Grande Ospedale Metropolitano Niguarda, Milan, Italy; ^5^Rheumatology Unit, Department of Internal Medicine, ASST-Settelaghi, “Ospedale di Circolo - Fondazione Macchi”, Varese, Italy; ^6^Rheumatology Unit, University of Pisa, Pisa, Italy; ^7^Unit of Clinical Rheumatology, Department of Rheumatology and Clinical Sciences, ASST Centro Traumatologico Ortopedico G. Pini - CTO, Milan, Italy; ^8^Department of Rheumatology, Medical University of Graz, Graz, Austria; ^9^Department of Rheumatology, Hospital of Bruneck, Bruneck, Italy; ^10^Rheumatology Unit, Department of Clinical and Molecular Sciences, Carlo Urbani Hospital, Polytechnic University of Marche, Ancona, Italy; ^11^Rheumatology Unit, Ospedale Civile Maggiore, University of Verona, Verona, Italy; ^12^Department of Clinical and Molecular Medicine, S. Andrea University Hospital, “Sapienza” University, Rome, Italy; ^13^Department of Medical and Biological Science, Rheumatology Clinic, Azienda Sanitaria Universitaria Integrata, Udine, Italy; ^14^Academic Rheumatology Centre, Università degli Studi di Torino, Turin, Italy

**Keywords:** early arthritis, ultrasonography, diagnosis, systematic review, imaging

## Abstract

**Background:** Differential diagnosis in early arthritis is challenging, especially early after symptom onset. Several studies applied musculoskeletal ultrasound in this setting, however, its role in helping diagnosis has yet to be clearly defined. The purpose of this work is to systematically assess the diagnostic applications of ultrasonography in early arthritis in order to summarize the available evidence and highlight possible gaps in knowledge.

**Methods:** In December 2017, existing systematic literature reviews (SLR) on rheumatoid arthritis (RA), osteoarthritis (OA), psoriatic arthritis (PsA), polymyalgia rheumatica (PMR), calcium pyrophosphate deposition disease (CPPD), and gout were retrieved. Studies on ultrasound to diagnose the target conditions and detecting elementary lesions (such as synovitis, tenosynovitis, enthesitis, bone erosions, osteophytes) were extracted from the SLRs. The searches of the previous reviews were updated and data from new studies fulfilling the inclusion criteria extracted. Groups of reviewers worked separately for each disease, when possible diagnostic accuracy (sensitivities, specificities) was calculated from primary studies. When available, the reliability of ultrasound to detect elementary lesions was extracted.

**Results:** For all the examined disease, recent SLRs were available. The new searches identified 27 eligible articles, with 87 articles included from the previous SLRs. The diagnostic performance of ultrasound in identifying diseases was addressed by 75 studies; in most of them, a single elementary lesion was used to define diagnosis, except for PMR. Only studies on RA included consecutive patients with new onset of arthritis, while studies on gout and CPPD often focused on subjects with mono-arthritis. Most of the remaining studies enrolled patients with a defined diagnosis. Synovitis was the most frequently detected lesion; clinical diagnosis was the most common reference standard. The diagnostic performance of ultrasound across different conditions was extremely variable. Ultrasound to identify elementary lesions was assessed in 38 studies in OA, gout and CPPD. Its performance in OA was very variable, with better results in CPPD and gout. The reliability of ultrasound was moderate to good for most lesions.

**Conclusions:** Although a consistent amount of literature investigated the diagnostic application of ultrasound, in only a minority of cases its additional value over clinical diagnosis was tested. This SLR underlines the need for studies with a pragmatic design to identify the placement of ultrasound in the diagnostic pathway of new-onset arthritis.

## Introduction

With effective treatment strategies for inflammatory arthropathies becoming extensively available, in the last decade a prompt diagnosis, allowing intervention within the window of opportunity, has become a critical point in the management of early arthritis (1). However, in rheumatology diagnosis can be achieved with certainty in a minority of cases, and this is particularly true when patients are assessed at very early stages of diseases. While in some cases the presence of valuable biomarkers, such as anticyclic citrullinated peptides antibodies (ACPA), drives the diagnostic process, in seronegative early arthritis the degree of uncertainty remains high. Moreover, the current classification criteria for the main rheumatic diseases, which are often inappropriately used to help diagnosis, require differential diagnosis to be performed before they are applied (2). This difficulty in the correct definition of diagnoses at early stages might lead to inappropriate management, delaying the start of effective treatment but also exposing patients to useless and potentially toxic drugs. In addition, also in a research setting, an imprecise diagnosis implies the impossibility to measure reliably the effect of innovative treatments in early phases. In this context, there is a great interest in the research of new biomarkers and new tools to help the diagnostic process.

Musculoskeletal ultrasonography has been widely applied in rheumatic diseases, demonstrating to be a valid and reproducible tool in both inflammatory and non-inflammatory pathologies. The relevance of this instrument has also been recognized by the European League Against Rheumatism (EULAR), that recommends ultrasound among the imaging which can be considered to help the clinical management of several conditions (3–5). The applications of ultrasound cover the areas of diagnosis, assessment of prognosis, follow-up of diseases and guide for intra-articular and peri-tendinous procedures. In the field of diagnosis, most of the studies on ultrasound investigated the frequency of elementary lesions characteristics of diseases, thus providing information on the diagnostic performance of this tool to detect single abnormalities or on the performance of single lesions to diagnose a disease. On the other hand, only a minority of studies tests the diagnostic value of combinations of lesions, assessed at the same time. Moreover, in this context elementary lesions are not selected based on their diagnostic properties and specificity for a certain condition. Only a minority of studies, in which the added value of ultrasound is tested jointly with clinical evaluation (6, 7), apply a pragmatic design that reproduces the clinical context. The lack of information on the application of ultrasound in a realistic clinical process of diagnosis translates into the limited weight given to this imaging in classification criteria. For instance, the only role for ultrasound in Rheumatoid Arthritis (RA) classification is the possible confirmation of the presence of synovitis (2), while to date the only classification criteria including ultrasound are those for polymyalgia rheumatica (PMR) (8).

Given the limited availability of methodologically sound studies to address the diagnostic performance of ultrasound in a realistic clinical context of differential diagnosis of inflammatory arthropathies, the Ultrasound Study Group of the Italian Society for Rheumatology (SIR) prioritized its research on this subject. The present study represents the first step of such project. The aim of the present work was the evaluation of the available literature on the diagnostic application of ultrasound in inflammatory arthropathies.

## Materials and Methods

As a first step, the most relevant differential diagnoses in patients with suspected inflammatory arthropathies were identified, including also osteoarthritis (OA) as a relevant differential diagnosis. We afterwards individuated two research questions, rephrased following the PICOs (Patient, Intervention, Comparator, Outcome, Study type) methodology to provide inclusion and exclusion criteria (Table 1). On this basis, we planned separate systematic literature reviews (SLR) to assess the diagnostic performance of ultrasound to diagnose OA, RA, psoriatic arthritis (PsA), PMR, gout, calcium pyrophosphate deposition disease (CPPD). The SLRs were not registered, but a common protocol was available for all researchers before the beginning of the process. The diagnostic performance of ultrasound in detecting elementary lesions was also addressed. If studies on diagnostic performance reported also data on intra and inter-reader reliability on elementary lesions, that information was also extracted. Working groups composed by supervisors and fellows were created to work separately on each topic, participants were selected based on the expertise on the specific disease and on SLR methodology to create uniform groups. The most recent SLRs on ultrasound in the same diseases were first sought in electronic databases (5, 9–13). Some of the authors involved in the present project were also co-authors of these SLR and could provide background material (AA, ABa, AI, AZ, CS, EF, GF, GS). Since many of the existing SLR had a broader focus, only primary studies focusing on the diagnostic use of ultrasound were taken into account for the present work.

**Table 1:** Inclusion criteria for research questions.

	**Population**	**Intervention**	**Comparators (reference standard)**	**Outcomes**	**Study type**
What is the added value of ultrasound to diagnose the target diseases?	People presenting with joint symptoms	Ultrasound	Clinical diagnosis (without imaging) Other imaging	Confirmation of the diagnosis	Systematic literature reviews, meta-analyses, RCTs, controlled trials, non-controlled trials, diagnostic accuracy studies, cohort studies, cross-sectional studies, case-control studies
What is the accuracy of ultrasound for detecting elementary lesions of the target diseases?	Patients with confirmed diagnosis of the target disease	Ultrasound	Physical examination Surgery Other imaging	Sensitivity, specificity, Likelihood ratios, Diagnostic Odds Ratio, AUC, negative predictive value, positive predictive value Inter-reader and intra-reader reliability	Systematic literature reviews, meta-analyses, RCTs, controlled trials, non-controlled trials, diagnostic accuracy studies, cohort studies, cross-sectional studies, case-control studies

The search strategies of the previous SLR were applied in PubMed and Embase, starting from the date of the last search of the previous reviews (5, 9–13). Searches were last run on November 30th 2017. The search on PubMed and Embase was selected because we expected that all the relevant literature would be retrieved, and we did not expect to find further evidence including other databases. The records retrieved from the new searches were transferred into a bibliographic manager software (Zotero, RRID:SCR_013784) and libraries shared with each working group. The titles and abstracts of the retrieved records were evaluated by pairs of reviewers to assess the eligibility for full-text review according to the pre-specified criteria. Full-texts were afterwards evaluated by the same criteria and data from the included studies extracted into a standardized form, including 2 × 2 tables of diagnostic performance. A flow-chart describing the selection process was separately generated for each SLR. Results were summarized through summary of findings tables, describing both studies included in the previous reviews and those identified by the present ones.

## Results

In total, all search strategies retrieved 943 references since the date of the last search of the previous SLRs. The higher number of references belonged to the fields of PsA and gout (Additional Online File). After reviewing the abstracts, 27 papers were finally included, together with 87 articles from previous SLRs meeting the inclusion criteria, for a total of 114 papers included in the present SLR (Table 2). The PRISMA flow-chart of the SLR for each disease is available in the Additional Online File, as well as the full results, presented through summary of findings tables.

**Table 2:** Features of the SLRs used as a basis for the present work.

**Target disease**	**References**	**Aim of the SLR**	**Last searches**	**Number of studies included in the present SLR**
RA	(12)	To evaluate the added value of ultrasound over clinical findings to the diagnosis of RA in patients with suspected arthritis	November 2015	11
OA	(5)	To provide evidence for the development of the EULAR recommendations for the use of imaging for the clinical management of OA. The SLR does not focus only on ultrasound	December 2015	18
PsA	(9)	To provide evidence for the selection and design of an observational study of the Ultrasound Study Group of the SIR. The SLR focuses on ultrasound	September 25^th^ 2015	24
CPPD	(10)	To provide evidence on the diagnostic performance of ultrasound to diagnose CPPD and to retrieve all the ultrasound definitions of CPPD. The SLR focuses on ultrasound.	31 December 2014	18
PMR	(13)	To review the accuracy of imaging to diagnose PMR	October 2^nd^ 2013	10
Gout	(11)	To provide evidence on the diagnostic performance of ultrasound to help clinicians in the choice of imaging. The SLR does not focus only on ultrasound	February 2016	6

### Ultrasound for the Clinical Diagnosis of Inflammatory Arthropathy

Information regarding the value of ultrasound to diagnose diseases could be extracted from 75 studies. The greatest amount of evidence was available for PsA, with 29 studies assessing the diagnostic performance of ultrasound.

There were meaningful differences in terms of enrolled populations across different diseases. In fact, in studies addressing PsA and OA, the primary aim was mostly to report the prevalence of different lesions. The frequency of each lesion was compared in patients with already known PsA or OA and healthy controls or patients with other definite diseases. A realistic clinical scenario of consecutive patients referred for suspicion of inflammatory arthropathy was rarely available (6).

Conversely, studies on RA evaluated the added value of ultrasound over classification criteria (14–17), the added value for diagnosis on top of clinical findings (18–20) or its prognostic value over the future development of RA (21–25) by cross-sectional or longitudinal study design.

Studies dealing with PMR mostly included populations of consecutive patients with shoulder pain (8, 13, 26) and some of them evaluated the additional value of ultrasound on the diagnostic performance of the 2012 classification criteria (8, 26, 27).

In the fields of both gout and CPPD, most of the studies included patients presenting with mono-arthritis and with suspect crystal-related arthritis.

Despite these discrepancies across different conditions, there were only a few studies, mainly focused on RA, that enrolled a population of consecutive patients with joint pain (6, 16–20, 22, 24, 28).

**Graph 1:**
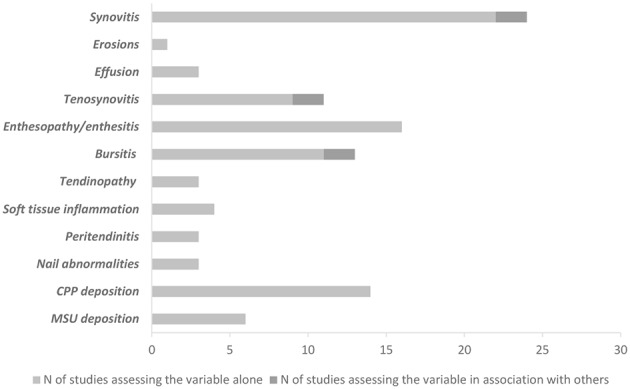
Number of studies using each single elementary lesion to establish diagnoses, alone or in combination. CPP, calcium pyrophosphate; MSU, monosodium urate.

The interventions used to help diagnosis were also variable. Since most of the studies did not have diagnostic accuracy as primary objective, data on the diagnosis of disease were based on single elementary lesions. A relevant exception was represented by PMR, for which some studies addressed different lesions (tenosynovitis, bursitis and synovitis) in combination (8, 26, 27). Graph 1 summarizes all the different lesions used to define diagnosis.

**Graph 2:**
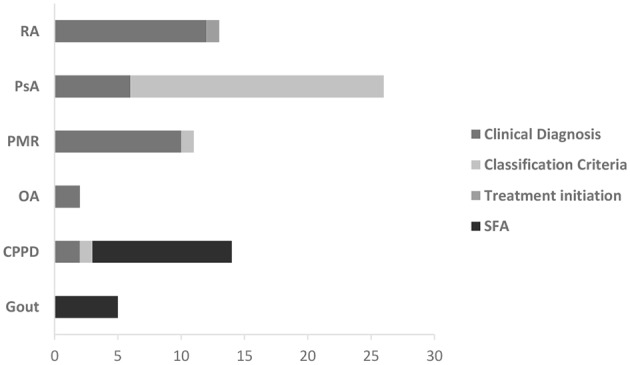
Reference standards adopted to confirm diagnoses. PMR, polymyalgia rheumatica; CPPD, calcium pyrophosphate deposition disease; OA, osteoarthritis; PsA, psoriatic arthritis; RA, rheumatoid arthritis; SFA, synovial fluid analysis.

The confirmation of the diagnosis was based on a variety of reference standards, which depended on the diagnostic suspicion, as expected. While clinical diagnosis was frequently considered in RA and PMR, for PsA the confirmation of diagnosis mostly relied on clinical diagnosis and classification criteria, while synovial fluid analysis was frequently considered in crystal-related arthropathies (Graph 2).

**Graph 3:**
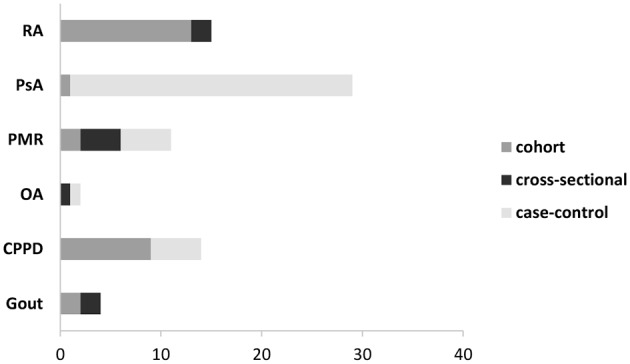
Study design of the included studies, depending on the assessed disease. PMR, polymyalgia rheumatica; CPPD, calcium pyrophosphate deposition disease; OA, osteoarthritis; PsA, psoriatic arthritis; RA, rheumatoid arthritis; SFA: synovial fluid analysis.

The study design adopted to define diagnostic accuracy was also widely variable. While studies aiming to diagnose RA were mainly cohort studies (15–24, 28), in the field of PsA emerged a significant prevalence of studies with a case-control design; controls were represented mostly by patients with RA (29–37), while in some studies also healthy controls were included (29, 31, 33, 38–43). For the remaining diseases, the type of study was more variable (Graph 3).

In OA, adding ultrasound information to the clinical evaluation increased the certainty of the diagnosis made by the clinician (6), while the likelihood of OA, compared to being healthy, increased with the finding of bone erosions (44).

In the field of RA, some studies supported the possibility to integrate clinical and ultrasound findings to reclassify undifferentiated arthritis (14–17, 21), while in other studies ultrasound information was applied to confirm a diagnosis of RA or tested against a clinical diagnosis (18–20), leading in general to an increase in diagnostic performance. The prognostic value of ultrasound in predicting the future development of the disease or the need for specific treatment has also been tested, once again with positive results supporting this application (22–24, 28) (Tables 3, 4). The most specific lesion to diagnose RA were bone erosions, with the specificity of 1 reported by a single study (19), although also the specificity of PD positive synovitis was high (ranging from 0.88 to 0.93).

**Table 3:** Performance of ultrasound to detect RA by elementary lesions and reliability.

Lesion*	**Sensitivity**		**Specificity**		**Intra-reader kappa**		**Inter-reader kappa**	
	**Min**	**Max**	**Min**	**Max**	**Min**	**Max**	**Min**	**Max**
*Erosions*	–	0.38	–	1	–	0.93	–	–
*GS synovitis*	0.69	0.94	0.5	0.86	0.83	0.94	0.56	0.86
*PD synovitis*	0.72	0.89	0.88	0.93	0.87	0.99	0.64	0.89

Summary of sensitivities, specificities and reliability across studies assessing the performance of ultrasound to diagnose RA elementary lesions. Of the 13 papers included,only four reported sensibility-sensitivity by using gray scale (GS) and/or power Doppler (PD) ≥ 2. *Hands (including proximal interphalangeal joints) and wrists.

**Table 4:** Performance of ultrasound to predict RA by elementary lesions by site.

	**Sensitivity**	
**GS SYNOVITIS**	**Sensitivity**	**Specificity**
Wrist	0.79	0.69
MCP	0.9	0.48
PIP	0.79	0.66
**PD SYNOVITIS**		
Wrist	0.9	0.48
MCP	0.9	0.66
PIP	0.66	0.76

*Summary of sensitivities, specificities across studies assessing the performance of ultrasound to predict RA by elementary lesions by site. Only Filer reports sensitivity and sensitivity for every joint. Gray Scale and power Doppler ≥ 2. MCP, Metacarpophalangeal; PIP, Proximal interphalangeal*.

Despite a higher number of studies with a focus on PsA, in this area there was a greater variability, due to many different lesions, tissues and sites assessed. Many studies (14 studies) focused on the assessment of entheseal abnormalities (30, 34, 35, 39, 40, 42, 45–52) and the joints (6 studies) (29, 32, 33, 38, 41, 53), while only a few studies assessed the fingers (considering joints, tendons, soft tissues and entheses) (31, 36, 37) or the nails (43, 54, 55). The primary aim of the included studies rarely addressed the diagnostic accuracy. In fact, most of the studies compared the prevalence of lesions in PsA and other diseases. Also, for this, the diagnostic performance of ultrasound findings, which were usually considered alone and not in combination or in addition to clinical findings, was extremely variable across lesions and sites (Table 5). Among the tested lesions, those proving to be more specific to detect PsA were those at the level of the entheses. In fact, the specificity of entheseal PD ranged from 0.33 to 0.99, of enthesophytes from 0.52 to 1 and of calcifications from 0.86 to 0.97. Peritenonitis was also very specific (from 0.95 to 1 when PD signal was present).

**Table 5:** Performance of ultrasound lesions to detect PsA and reproducibility.

**Lesion**	**Sensitivity**		**Specificity**		**Intra-reader kappa**		**Inter-reader kappa**	
	**Min**	**Max**	**Min**	**Max**	**Min**	**Max**	**Min**	**Max**
Synovial hypertrophy	0.16	0.76	0	1	–	–	0.78–1	–
Joint effusion	0.07	0.61	0.33	0.82	–	–	–	–
Erosions	0.04	0.58	0.40	1	–	–	–	–
Enthesopathy	0.22	1	0.20	1	–	–	–	–
Entheseal PD	0.05	0.3	0.30	0.99	0.91	0.97	–	–
Entheseal erosions	0.05	0.20	0.96	1	–	–	–	–
Enthesophytes	0.15	0.55	0.52	1	–	–	–	–
Entheseal calcifications	0.02	0.19	0.86	0.97	–	–	–	–
Peritenonitis PD	0.36	0.65	0.95	1.00	–	–	–	–
Peritenonitis GS	0.54	0.60	0.95	0.97	–	–	–	–
Soft tissue oedema	0.29	0.42	0.90	1	–	–	–	–
Bursitis	0.02	0.10	0.90	0.99	0.96	–	0.87	–

*Summary of sensitivities, specificities and reliability across studies assessing the performance of ultrasound to diagnose PsA. Min, minimal; Max, maximal; PD, power Doppler; GS, gray scale*.

Studies focusing on ultrasound of the hips and the shoulders in PMR had a more variable design. In fact, along with some older studies with a case-control design (8, 56–59) several cohort studies, including that on which the current classification criteria are based (8), included consecutive patients with shoulder pain (34, 60). Moreover, several recent studies provided external validation for the classification criteria (26). Again, in terms of accuracy, studies yielded very heterogeneous results (Table 6). In general, bilateral findings seemed to be more specific for PMR. The specificity of bilateral subacromiodeltoid bursitis ranged from 0.68 to 0.99, while for bilateral long head of the biceps tenosynovitis ranged from 0.62 to 0.98.

**Table 6:** Performance of ultrasound lesions to detect PMR.

**Lesion**	**Sensitivity**		**Specificity**	
	**Min**	**Max**	**Min**	**Max**
SAD bursitis at least monolateral	0.09	0.96	0.59	0.90
SAD bursitis bilateral	0.32	0.93	0.68	0.99
LHB tenosynovitis at least monolateral	0.14	0.81	0.47	0.59
LHB tenosynovitis bilateral	0.30	0.37	0.62	0.98
GH synovitis at least monolateral	0.20	0.77	0.34	0.78
GH synovitis bilateral	0.03	0.52	0.66	0.90
Hip synovitis at least monolateral	0.24	0.45	0.55	0.88
Hip synovitis bilateral	0.18	0.38	0.83	0.92
Trochanteric bursitis at least monolateral	0.21	0.98	0.70	0.91

*Summary of sensitivities, specificities across studies assessing the performance of ultrasound to diagnose PMR. Min, minimal; Max, maximal; SAD, subacromiodeltoid; LHB, long head of the biceps; GH, gleno-humeral*.

Studies in CPPD evaluated several different sites, including the knees (61–68), the wrist (69, 70), the affected joint or all joints (71). Study design was variable, including both case-control and cohort studies. The diagnosis of CPPD was confirmed more frequently by synovial fluid analysis, while in some cases a clinical diagnosis (70, 71) or histology (68) were used as references. In general, ultrasound seemed to perform well in identifying this condition, especially at the knee and the wrist. The specificity to confirm CPPD at the knee (considering all the assessed sites) ranged from 0.66 to 1, while at the wrist from 0.81 to 0.91.

In the field of gout, the type of joint under investigation was widely variable, all studies (72–74) but two (75, 76) adopted synovial fluid analysis as reference standard to diagnose the disease. 4/6 studies had a cross-sectional design, while the two remaining were a prospective (73) and a retrospective (72) study. While 4 studies reported a satisfactory performance of ultrasound (73–75, 77), for 2 studies sensitivity was low (72, 76). Considering the combination of all possible elementary lesions (e.g., double contour, aggregates, tophi), the specificity of ultrasound to diagnose gout ranged from 0.42 to 0.87.

### Ultrasound to Diagnose Elementary Lesions

Data on the accuracy of ultrasound to detect elementary lesions were extracted only for OA, CPPD and gout, with 20 (78–96), 12 (68–70, 97–104), and 6 (105–110) studies addressing this aspect, respectively (Tables 7–10).

**Table 7:** Performance of ultrasound to detect osteoarthritis elementary lesions and reliability.

**Site/lesion**	**Sensitivity**		**Specificity**		**Intra-reader kappa**		**Inter-reader kappa**	
	**Min**	**Max**	**Min**	**Max**	**Min**	**Max**	**Min**	**Max**
Knee osteophytes								
Vs CR	0.95	0.99	0.57	0.94	0.82	0.87	–	–
Vs hist.	0.7	0.9	–	–				
Hand osteophytes								
Vs CR	0.83	0.96	0.65	0.76	0.087	1	0.53	0.69
Vs MRI	0.82	0.9	0.75	0.95				
Vs PE	0.89	–	0.68	–				
Foot osteophytes								
Vs CR	0.62	–	0.86	–	–	–	–	–
Hand JSN								
*Vs CR*	0.82	–	0.72	–	–	–	–	–
Knee cartilage damage								
Vs CR	1	–	1	–	–	–		–
Vs hist.	0.78	0.89	–	–			0.67	
Hand erosions								
Vs CR	0.73	0.94	0.90	1	0.81	–	0.69	0.90
Vs MRI	0.65	0.88	0.90	0.96				
Knee erosions								
Vs CR	0.33	–	0.99	–	–	–	–	–
Foot erosions								
Vs CR	0.33	–	0.98	–	–	–	–	–
Knee effusion								
Vs PE	0.74	1	0	0.52	–	–	–	–
Vs JA	1	–	0	–				
Vs MRI	0.81	–	–	–				
Hand effusion								
Vs PE	1	–	–	–	0.81	–	0.69	–
Vs MRI	0.92	–	0.98	–				
Popliteal cyst								
Vs PE	0.36	0.67	0.89	0.98	–	–	–	–
Vs scint.	0.29	–	0.90	–				
Hand cysts								
Vs MRI	0.87	–	0.97	–	0.81	–	0.69	–
Hand synovitis								
Vs PE	0.15	–	0.96	–	0.81	–	0.69	–
Vs MRI	0.84	–	0.96	–				
Knee synovitis								
Vs PE	0.67	–	0.50	–	–	–	–	–
Pes anserinus bursitis								
Vs PE	0.50	–	0.96	–	–	–	–	–

*Summary of sensitivities, specificities and reliability across studies assessing the performance of ultrasound to diagnose OA elementary lesions. Min, minimal; Max, maximal; CR, conventional radiography; hist, histology; MRI, magnetic resonance imaging; PE, physical examination; JA, joint aspiration; scint, scintigraphy*.

**Table 8:** Performance of ultrasound to detect CPPD elementary lesions and reproducibility.

**Site/lesion**	**Sensitivity**		**Specificity**		**Intra-reader kappa**		**Inter-reader kappa**	
	**Min**	**Max**	**Min**	**Max**	**Min**	**Max**	**Min**	**Max**
Knee FC	0.007	0.96	0.50	1.00	–	–	0.68	0.81
Knee HC	0.59	1.00	0.00	1.00	–	–	0.55	0.81
Wrist TFCC	0.78	0.81	0.85	0.91	–	–	–	–

*Summary of sensitivities, specificities and reliability across studies assessing the performance of ultrasound to diagnose CPPD elementary lesions. FC, fibrocartilage; HC, Hyaline Cartilage; TFCC, Triangular Fibrocartilage Complex*.

**Table 9:** Performance of ultrasound to detect gout elementary lesions and reproducibility (acute attack).

**Site/lesion**	**Sensitivity**		**Specificity**		**Intra-reader kappa**		**Inter-reader kappa**	
	**Min**	**Max**	**Min**	**Max**	**Min**	**Max**	**Min**	**Max**
Knee/DC	–	0.75	–	–	–	–	–	–
Knee/tophi	—	0.62	–	–	–	–	–	–
1st MTP/DC	0.62	0.87	–	–	–	–	–	–
1st MTP/tophi	0.71	0.87	–	–	–	–	–	0.82
1st MTP/erosion	0.52	–	–	–	–	–	–	0.83
1st MTP/effusion	0.29	–	–	–	–	–	–	–
Knee/1st MTP erosion	0.31	0.48	0.53	0.79	–	–	–	–
Knee/1st MTP DC	0.34	0.51	0.91	0.99	–	–	–	0.87
Knee/1st MTP tophi	0.21	0.65	0.96	1.0	–	–	0.47	0.83
Knee/1st MTP echogenic foci	0.71	0.85	0.56	0.73	–	–	–	–
Symptomatic joint or tendon/erosion	0.11	0.33	0.03	0.81	1.0	–	0.86	–
Symptomatic joint or tendon/hypervascularization	0.88	0.98	0.39	0.66	0.83	-	0.67	–
Symptomatic joint or tendon/HCA	0.25	0.87	0.18	0.99	0.81	–	0.58	0.71
Symptomatic joint or tendon/DC	0.36	0.52	0.83	0.96	1.0	–	0.63	0.71
Symptomatic joint or tendon/tophi	0.29	0.52	0.95	1.0	–	–	0.74	–

*Summary of sensitivities, specificities and reliability across studies assessing the performance of ultrasound to diagnose gouty elementary lesions (acute attack). Min, minimal; Max, maximal; MTP, metatarsophalangeal; DC, double contour; HCA, hyperechoic cloudy area*.

**Table 10:** Performance of ultrasound to detect gout elementary lesions and reproducibility (intercritic phase).

**Site/lesion**	**Sensitivity**		**Specificity**		**Intra-reader kappa**		**Inter-reader kappa**	
	**Min**	**Max**	**Min**	**Max**	**Min**	**Max**	**Min**	**Max**
Knee effusion	0.92	1.0	0.77	0.95	–	–	–	–
Knee synovial hypertrophy	0.49	0.74	0.92	1.0	–	–	–	–
Knee intra-articular PD	0.20	0.45	0.92	1.0	–	–	–	–
Midtarsal joints /effusion, synovial hypertrophy, erosion, tophi	0.91	0.94	0.93	0.96	–	–	–	–
MTP joints/effusion, synovial hypertrophy, erosion, tophi	0.90	0.95	0.78	0.85	–	–	–	–
Multiple sites/intra-articular or intrabursal HAG	0.78	0.91	0.65	0.91	–	0.67	0.50	0.54
Tendon/ligament HAG	0.55	0.72	0.84	0.95	–	0.67	0.50	0.54
Tendon/hyperechoic linear band	0.47	0.64	0.65	0.91	–	0.70	0.35	0.36
Cartilage/DC	0.66	0.82	0.76	0.89	–	0.88	0.69	0.74
1st MTP erosion	0.51	0.77	0.84	0.98	–	–	0.29	0.74
1st MTP DC	0.53	0.84	0.59	1.0	–	–	0.37	0.61
1st MTP tophi	0.26	0.77	0.88	1.0	–	–	0.26	0.78
1st MTP effusion	0.09	0.30	0.51	0.77	–	–	0.23	0.60
1st MTP synovial hypertrophy	0.03	0.19	0.92	1.0	–	–	0.36	0.81
1st MTP synovitis	0.01	0.14	0.73	0.93	–	–	0.48	0.83

*Summary of sensitivities, specificities and reliability across studies assessing the performance of ultrasound to diagnose gouty elementary lesions (intercritic phase). Min, minimal; Max, maximal; PD, power Doppler; MTP, metatarsophalangeal; DC, double contour; HAG, hyperechoic aggregate*.

The typical population enrolled was represented by subjects with confirmed disease, in which ultrasound was compared to a reference standard to confirm the presence of a lesion.

As expected, also the reference standard was variable, in particular for OA. For CPPD, the only assessed target lesion was CPP deposition, which was evaluated by conventional radiography (2 studies), synovial fluid analysis (6 studies), microscopic analysis (2 studies). All studies on gout but one (107), in which conventional radiography was used, adopted synovial fluid analysis as reference standard.

Most of the studies assessing ultrasound to detect elementary lesions had a cross-sectional design, in particular, all the studies on OA, 4 (74–77) and 9 (62, 69, 70, 99–101, 103) studies for gout and CPPD, respectively, while the remaining studies for these two conditions had a cohort design.

In OA, results on the performance of ultrasound were once again widely variable across studies. This was also due to the variability of the reference standards adopted to define each separate lesion and the assessment of different anatomical areas.

Most of the studies on CPPD reported good performance of ultrasound to detect deposits, and this was true especially for specificity. The same conclusions can be drawn from the included articles on gout.

### Reliability

Most of the studies on OA in which reliability data were presented reported good reliability for the assessment of osteophytes, erosions, effusion, cartilage damage, synovitis and cysts (Table 7).

In RA, the available evidence supported a good intra-reader and inter-reader reliability for erosions, GS and PD synovitis across all the assessed sites (Tables 3, 4).

There was less information about reliability in the ultrasound assessment of PsA; entheseal PD, synovial hypertrophy and bursitis were the only lesions for which reliability was available. Inter-reader reliability was good for synovial hypertrophy and bursitis, as well as intra-reader for entheseal PD and bursitis (Table 5).

Among the included studies on PMR, none reported information on the reliability for the assessed lesions.

For CPPD, some studies reported a good inter-reader reliability to assess both the meniscal fibrocartilage and the hyaline cartilage at the level of the knee (Table 8). In gout during acute attacks, very good intra-reader reliability was reported for double contour, aggregates, erosions and hypervascularisation. Inter-reader reliability was assessed for tophi, erosions, double contour, hypervascularisation and aggregates, still with good values (Table 9). The reliability on the same lesions was also assessed in the intercritical phases, with still good, although in general lower, results (Table 10).

## Discussion

The aim of our SLR was that of retrieving all the available evidence to support future studies on the integration of the information provided by ultrasound in the diagnostic process. Several groups had already focused on this aspect, since recent SLRs were available for all of the conditions of our interest (5, 9, 11, 13, 104). The existing reviews presented a summary of the diagnostic use of ultrasound deriving from a relevant number of studies for each considered disease. Despite all the reviews being relatively recent, we found additional studies in the subsequent literature from which we could retrieve further evidence. The number of SLRs and eligible studies represents a clue of the interest that ultrasound as diagnostic tool has raised. The easier availability of high-end ultrasound equipment, the accessibility to training and the possibility to apply directly the information provided by ultrasound during a routine visit are likely the features that have driven the enthusiasm about the technique. However, when analyzing in depth the available literature, there is an evident gap between the interest in the diagnostic applications of ultrasound and the quality of the studies produced so far in this field. In fact, with some important exceptions, the main objective of the studies was that of describing the prevalence of different lesions and comparing groups of patients in terms of ultrasound findings. Although information on diagnostic accuracy can be retrieved also from such study design, these results cannot be generalized to external populations, since a realistic clinical setting is not reproduced.

Many studies, in fact, included patients with definite and longstanding diagnosis and adopted a case-control design, with controls that were unlikely to be very similar to the true differential diagnoses of disease. This is particularly true for PsA, for which most of the studies had a case-control design.

There was limited evidence regarding the diagnosis of OA (6), while for RA and PMR the studies reproduced a more pragmatic context. In fact, in RA, some studies evaluated patients with new-onset arthralgia and tested the ability of ultrasound to help confirm diagnosis (19), while some others integrated ultrasound on top of classification criteria (16, 17). There were also some studies testing the prognostic value of ultrasound on the future development of RA (22).

In the context of PMR, some older studies still adopted a case-control design (56), however, since the development of the new classification criteria (8), the interest has shifted to the evaluation of the additional impact of ultrasound on classification (26). The performance of US in this context was highly variable. Such heterogeneous results might be due to the disease, which may present with variable abnormalities, thus affecting the US sensitivity. Bilateral pathologic conditions appear to be the most specific US findings.

In the field of crystal-related arthropathies, several studies evaluated both patients during the acute presentation and the inter-critical periods. The population of interest was that of patients presenting with monoarthritis, representing a realistic clinical scenario for this diagnostic suspicion, although quite specific.

The ability of ultrasound to correctly identify elementary lesions typical of each disease seemed to be good, and this was especially true for inflammatory lesions. When a suboptimal performance was achieved, it must be kept in mind that in several studies the reference standard adopted to define a lesion (e.g., physical examination) could not be considered the optimal one for the specific lesion.

Although this was not the primary objective of this SLR, we extracted information on intra and inter-reader reliability, if available. The information from the primary studies supported good reliability of ultrasound to identify inflammatory lesions, as well as signs of damage, at the level of joints and entheses, as well as deposition of crystals. It must however be considered that rheumatologists taking part in ultrasound studies might have greater expertise on a specific lesion or disease than average, so that such reliabilities could not be reproduced in a clinical setting.

The present SLR has some limitations. First, only two databases were searched, and, although probably the greatest part of the literature has been covered, we cannot exclude the presence of further studies, even among gray literature. Due to the clinical heterogeneity of the results, we did not perform a pooled estimate of the diagnostic performance. Moreover, a formal assessment of quality and risk of bias was not performed. However, the present work is, to our knowledge, the first one to provide a comprehensive overview on the diagnostic use of ultrasound in arthritis, with a focus on the general question and without concentrating on a single disease.

What emerges from the overview of the results of our SLR is that a very few studies (6, 16, 19, 22, 24) investigated the additional impact of ultrasound findings in making a diagnosis in consecutive patients presenting with joint symptoms, which is indeed the typical scenario of every day's rheumatologist work.

In most studies, clinical and ultrasound assessments were performed separately, and ultrasound findings were not evaluated on top of clinical findings but validated against clinical diagnosis. With this being almost the only evidence available today, it is of no surprise that so far, the relevance of ultrasound in recommendations on the diagnosis and management of rheumatic diseases and in classification criteria is so limited. This happens despite ultrasound being an ideal tool in this context: adequate ultrasound equipment can now be easily accessible, they can be used during scheduled visits and provide immediately helpful information. Multiple sites can be assessed at the same time with good acceptability by the patients. Several other modern imaging have been applied in the setting of early arthritis, such as magnetic resonance imaging (MRI), positron emission tomography (PET) od dual energy CT (DECT), however they present a limited feasibility compared to ultrasound, limited availability, higher costs and, in some cases, limited data in the clinical setting. Since the accuracy of ultrasound in detecting elementary lesions has been established and the increasing ultrasound expertise across rheumatologists allows at least some findings to be detected reliably, the time has come to test the real potentialities of ultrasound during the first evaluation for the suspicion of inflammatory arthropathy. The Musculoskeletal ultrasound Study Group of the Italian Society for Rheumatology has recently focused on the design of such study, which implies the definition of the ideal combination of joints to be assessed based on the clinical suspicion and confirming diagnoses after a follow-up. Before the application of ultrasound, an initial set of differential diagnoses should be defined for each patient, based on clinical features. The additional value of an ultrasound examination, targeted on the clinical suspicion, would afterwards be tested in terms of correct and timely diagnosis. We expect that these results will help clarify the real role of ultrasound through the process of diagnosis and help giving a new insight into its correct placement in the management of inflammatory arthropathies.

## Data Availability Statement

The datasets generated for this study are available on request to the corresponding author.

## Author Contributions

GS, SC, GF, and AI conceived the study and supervised its conduction. SC performed the searches in the electronic databases. AA, ABa, ABo, AD, OD, CD, OE, EF, LI, AP, AZ, and GS performed the systematic literature review. GS drafted the manuscript. All authors revised critically the article, read and approved its final version for submission.

## Conflict of Interest

The authors declare that the research was conducted in the absence of any commercial or financial relationships that could be construed as a potential conflict of interest.
