# Supplementary material for: Differential Diagnosis of Inflammatory Arthropathies by Musculoskeletal Ultrasonography: A Systematic Literature Review
Source: Front Med (Lausanne). 2020 May 7;7:141. doi: 10.3389/fmed.2020.00141 (PMC7221062; doi:10.3389/fmed.2020.00141)
Supplement: Supplementary file 1 [file Table_1.DOCX]

Differential diagnosis of inflammatory arthropathies by musculoskeletal ultrasonography: a systematic literature review.

**Supplementary online material.**

***Osteoarthritis***

***Flow-chart§***

***
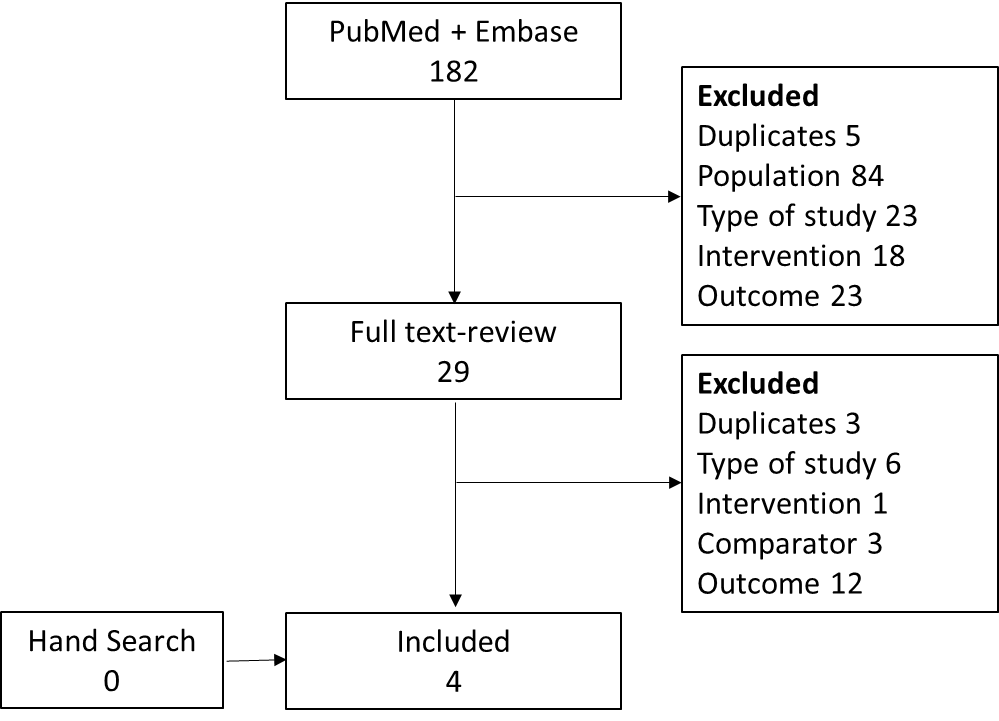
***

*§ 18 studies were retrieved from the previous SLR*

***Summary of findings table: US to diagnose OA elementary lesions***

Summary of findings of studies assessing the performance of US to diagnose OA elementary lesions. Estimates of diagnostic performance are presented as point estimate and 95% confidence intervals, unless specified. Se: sensitivity; Sp: specificity, LR+: positive likelihood ratio; LR-: negative likelihood ratio; MCP: metacarpophalangeal joints; CMC: carpometacarpal joint; PIP: proximal interphalangeal joint; DIP: distal interphalangeal joints; JSN: joint space narrowing; MRI: magnetic resonance imaging; PE: physical examination; CR: conventional radiography; US: ultrasonography; CEUS: contrast-enhanced ultrasonography; KLG: Kellgren and Lawrence grade; PPV: positive predictive value; NPV: negative predictive value.

| **Study** | **N** | **Site** | **Study design** | **Comparator/reference standard** | **Outcome** | | |
| --- | --- | --- | --- | --- | --- | --- | --- |
|  |  |  |  |  |  | **Diagnostic performance** | **Reliability** |
| Iagnocco A 2005(1) | 110 | Hand (PIP and DIP) | Cross-sectional cohort | Conventional radiography | Erosions | Se 0.72 (0.49,0.89)  Sp 1 (0.95,1)  LR- 0.27 (0.14,0.54) | Interobserver variation was 5% (nonsignificant) |
| Keen HI 2008(2) | 37 | Hand (CMC, MCP, PIP and DIP) | Cross-sectional cohort | Conventional radiography | - Osteophytes - Joint space narrowing | **Osteophytes:**  Se 0.83 (0.78,0.87)  Sp 0.75 (0.72,0.78)  LR + 3.42 (2.99,3.90)  LR - 0.25 (0.19,0.32)  **JSN:**  Se 0.82 (0.77,0.86)  Sp 0.72 (0.69,0.75)  LR + 2.92 (2.62,3.35)  LR - 0.22 (0.17,0.28) | **Intra-reader reliability**  Synovitis: kappa 0.074-1  Doppler: kappa 0.21-1  Osteophytes: kappa 0.087-1  **Inter-reader reliability**  Synovitis: kappa 0.398  Doppler: kappa 0.327  Osteophytes: 0.530 |
| Koutroumpas AC 2010(3) | 18 | Hand (PIP and DIP) | Cross-sectional cohort | Physical examination | Synovitis (GS and PD) | With PD as reference standard for inflammation, the Se of clinical assessment was 0.15 and the Sp 0.96 | n.a. |
| Mathiessen A 2013(4) | 127 | Hand (CMC, MCP, PIP and DIP) | Cross-sectional cohort | Physical examination  Conventional radiography  MRI | Osteophytes | **Vs PE:**  Se 0.89 (0.87,0.91)  Sp 0.68 (0.67,0.70)  LR+ 2.89 (2.71,3.08)  LR- 0.15 (0.13,0.18)  **Vs CR:**  Se 0.04 (0.029,0.05)  Sp 0.35 (0.33,0.36)  LR + 0.06 (0.05,0.08)  LR- 2.74 (2.59,2.90)  **Vs MRI**:  Se 0.95 (0.93,0.96)  Sp 0.86 (0.81,0.90)  LR + (5.04,9.93)  LR- 0.05 (0.04,0.08) | n.a. |
| Vlychou M 2009(5) | 22 | Hand (CMC, MCP, PIP and DIP) | Cross-sectional cohort | Conventional radiography | - Osteophytes - Erosions | Erosions were detected in 231/660 (35%) joints by US and in 115/660 (17.4%) joints with conventional radiographs [P<0.05]. . Osteophytes were detected in 360/660 (54.5%) joints by US imaging and in 310/660 (47%) joints with conventional radiographs [P<0.05] | **Intra-reader reliability**  kappa 0.81 |
| Vlychou M 2013(6) | 20 | Hand ( MCP, PIP and DIP) | Cross-sectional cohort | MRI | - Osteophytes - Erosions - Cysts - Synovitis - Tenosynovitis - Effusion | **Osteophytes:**  Se 0.9 (0.84,0.94)  Sp 0.95 (0.85,0.99)  **Erosions:**  Se 0.88 (0.81,0.93)  Sp 0.96 (0.89,0.98)  **Cysts:**  Se 0.87 (0.75,0.94)  Sp 0.97 (0.92,0.99)  **Synovitis:**  Se 0.84 (0.75,0.9)  Sp 0.96 (0.91,0.99)  **Tenosynovitis:**  Se 0.8 (0.57,0.94)  Sp 0.99 (0.96–1)  **Effusion:**  Se 0.92 (0.73,0.99)  Sp 0.98 (0.95,0.99) | **Intra-reader reliability**  kappa 0.81  **Inter-reader reliability**  Kappa 0.69 |
| Wittoek R 2010(7) | 38 | Hand (PIP and DIP) | Cross-sectional cohort | Conventional radiography | Erosions | Se 0.94(0.86,0.98)  Sp 0.90 (0.87,0.93) | n.a. |
| Wittoek R 2011(8) | 14 | Hand (PIP and DIP) | Cross-sectional cohort | MRI | Erosions | Se 0.65 (0.50,0.79)  Sp 0.90 (0.80,0.96)  LR + 10.20 (7.68,13.55)  LR – 0.06 (0.02,0.16) | **Inter-reader reliability**  kappa 95% CI: Bone erosions 0.90 (0.83 to 0.97) ; Osteophytes 0.83 (0.75 to 0.91); synovitis 0.93 (0.86 to 1.0); effusion 0.84 (0.76 to 0.92) |
| Iagnocco A 2000(9) | 57 | Hand (CMC) | Cross-sectional cohort | Joint aspiration | Joint effusion | Se 1 | n.a. |
| Akgul O 2014(10) | 110 | Knee | Cross-sectional cohort | Physical examination | Poplyteal cyst | Se 0.66 (0.47, 0.82)  Sp 0.98 (0.95,0.99)  LR + 42.22 (13.36, 133.45)  LR- 0.34 (0.20,0.56) | n.a. |
| Chatzopoulos D 2008(11) | 196 | Knee | Cross-sectional cohort | Radioisotope scanning Tc 99 | Poplyteal cyst | Se 0.28 (0.23,0.34)  Sp 0.90 (0.73,0.97)  LR + 2.89 (0.97,8.57)  LR - 0.79 (0.69,0.91) | n.a. |
| Esen S 2013 (12) | 100 | Knee | Cross-sectional cohort | Physical examination | - Joint effusion - Pes anserinus bursitis - Popliteal cyst | **Effusion**  Se 0.76 (0.56,0.92)  Sp 0.51 (0.40,0.63)  LR + 1.63 (1.19,2.24)  LR – 0.42 (0.19,0.94)  **Pes anserinus bursitis**  Se 0.50 (0.08,0.91)  Sp 0.95 (0.89,0.98)  LR + 12.25 (2.27,66.71)  LR – 0.52 (0.13,2.09)  **Poplyteal cyst**  Se 0.36 (0.23,0.50)  Sp 0.88 (0.75,0.96)  LR + 3.27 (1.33,8.03)  LR – 0.72 (0.57,0.90) | n.a. |
| Ike RW 2010 (13) | 14 | Knee | Cross-sectional cohort | Physical examination  Joint aspiration | Joint effusion | **Vs PE**  Se 1 (0.16,1)  SP 0 (0,0.19)  LR+ 1 (1,1)  **VS joint aspiration**  Se 1 (0.76,1)  Sp 0 (0,0.59)  LR + 1 (1,1) | n.a. |
| Lee CL 2008 (14) | 95 | Knee | Cross-sectional cohort | Histology | Cartilage damage | The in vivo US grading was significantly correlated to histologic grading over anterior and middle areas ( p<0.001, Rho=0.40 and 0.36) | **Inter-reader reliability**  Weighted kappa 0.67 |
| Pendleton 2008(15) | 86 | Knee | Cross-sectional cohort | Physical examination | - Joint effusion - Synovitis | **Effusion**  Se 0.73 (0.58,0.85)  Sp 0.15 (0.05,0.29)  LR+ 0.87 (0.70,1.08)  LR – 1.74 (0.72,4.21)  **Synovitis**  Se 0.67 (0.63,0.78)  Sp 0.50 (0.30,0.69)  LR + 1.34 (0.89,2.03)  LR - 0.66 (0.39,1.10) | n.a. |
| Song IH 2008 (16) | 41 | Knee | Cross-sectional cohort | MRI | Joint effusion **CEUS** | Se 0.72 – 0.81 | n.a. |
| Yoon CH 2008(17) | 51 | Knee | Cross-sectional cohort | Conventional radiography | Cartilage thickness | In longitudinal US scan, the maximum and minimum cartilage thicknesses of the medial femoral condyle, not the lateral condyle, showed significant correlations with the JSW (r=0.417, r=0.412 respectively, p<0.05; Pearson’s correlation coefficient) | n.a. |
| Okano T 2016(18) | 84 | knee | Cross-sectional cohort | Conventional radiography | - Osteophytes - Cartilage damage (0-3) | **Osteophytes:**  Se 0.95 (0.88,0.98)  Sp 0. 57 (0.41,0.91)  LR + 2.18 (1.56,3.04)  LR - 0.10 (0.04,0.23)  **Cartilage damage:**  Se 1 (0.97,1)  Sp 1 (0.16,1) | n.a. |
| Camerer M 2017(19) | 124 | Mid-foot | Cross-sectional cohort | Conventional radiography | - Osteophytes - Erosions | **Osteophytes**  Prevalence: US 14,1% vs CR 0,5%  Se 0.61 (0.31,0.86)  Sp 0.86 (0.84,0.87)  LR+ 4.45 (2.87,6.92)  LR- 0.45 (0.22,0.89)  **Erosions**  Prevalence: U 2.5% vs CR 0.1%  Se 0.33 (0.008,0.90)  Sp 0.99 (0.99,0.99)  LR+ 159.20 (25.69,986.71)  LR- 0.67 (0.30,1.49) | n.a. |
| Mortada M 2016(20) | 180 | knee | Cross-sectional cohort | Conventional radiography (KLG) | Osteophytes (0-4) | Diagnostic performance for osteophytes (categorical)  Se 0.99 (0.96,1)  Sp 0.94 (0.71,1)  LR + 16.79 (2.51,112.44)  LR – 0.01 (0.00,0.05)  KLG of CR as reference  (range of values for osteophytes grades)  US Se: 88.2-94.1  US Sp: 96.3-100  US PPV: 88.9-100  US NPV: 97.9-99.4  LR-: 0.02-0.08 | **Intra-reader reliability**  first reader:  89.4%, difference: 12.4%, kappa: 0.87, P ≤0.001  second reader: 86.5%, difference: 13.5%, kappa: 0.82, p ≤0.001.  **Inter-reader reliability**  detected grades: agreement: 82.3%, difference: 13.5%, kappa: 0.81, p ≤0.001 |

***Summary of findings table: US to diagnose OA***

Summary of findings of studies assessing the performance of US to diagnose OA. Estimates of diagnostic performance are presented as point estimate and 95% confidence intervals, unless specified. RA: rheumatoid arthritis; HC: healthy controls; PsA: psoriatic arthritis; OR: odds ratio; RR: risk ratio.

| **Study** | **N** | **Site** | **Study design** | **Comparator/reference standard** | **Population** | **Outcome** | | |
| --- | --- | --- | --- | --- | --- | --- | --- | --- |
|  |  |  |  |  |  |  | **Diagnostic performance** | **Reliability** |
| Matsos M 2009(21) | 62 | Hands and feet | Cross-sectional cohort | Clinical diagnosis | Consecutive patients referred for hands and feet US | Clinical diagnosis (Likert’s scale of the degree of confidence of clinical diagnosis) | **Certainty pre-US vs certainty post-US**  primary OA: 29 (46.8) vs 45 (73.0) p=0.002  inflammatory OA 29 (46.8) vs 54 (87.1) p<0.001 | n.a. |
| Zayat A 2015(22) | 60 OA  70 RA  60 PsA  60 gout  60 HC | Wrists and hands | Case-control | Clinical diagnosis | Patients with OA, RA, PsA, gout and healthy controls | Erosions vs Clinical diagnosis of OA | **Incident rate ratio (95% CI)**  RA vs OA 5.41 (3.92,7.47), p<0.001  PsA vs OA 2.16 (1.49,3.14), p<0.001  OA vs HC 2.16 (1.33,3.53), p 0.002  Maximum erosion diameter in any joint ≥ 2.5mm  OR 13.24 (1.65, 106.22) p 0.0150  RR: 11.18 (1.49,83.96) p 0.0189  Erosion score >0 any joint  OR 4.67 (2.16,10.08) p 0.0001  RR 2.18 (1.43,3.32) p 0.0003  Erosion score ≥ 2 any joint  OR 4.21 (0.48, 38.87) p 0.2044  RR 1.64 (1.02,2.65) p 0.0413 | **Intra-reader reliability**  Kappa 0.87 (0.79, 0.96)  0.82 (0.69 to 0.95)  **Inter-reader reliability**  Kappa 0.88 (0.76,1) |

***Rheumatoid Arthritis***

***Flow-chart§***


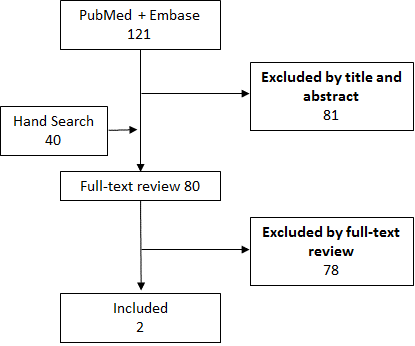


*§ 11 studies were retrieved from the previous SLR*

***Summary of findings table: US to diagnose RA***

Summary of findings of studies assessing the performance of US to diagnose RA. US: ultrasonography; GS: grey scale; PD: power Doppler; MRI: magnetic resonance imaging; Se: sensitivity; Sp: specificity; LR+: positive likelihood ratio; LR-: negative likelihood ratio; PPV: positive predictive value; NPV: negative predictive value; OR: odds ratio; HR: hazard ratio; MCP: metacarpophalangeal joints; PIP: proximal interphalangeal joints; DIP: distal interphalangeal joints; MTP: metatarsophalangeal joints.

| **Study** | **N** | **Site** | **Study design** | **Comparator/reference standard** | **Population** | **Outcome** | **Diagnostic performance** | **Reliability** |
| --- | --- | --- | --- | --- | --- | --- | --- | --- |
| Salaffi F 2010 (23) | 149 | 18 joints. Wrist, MCP 2–5, PIP 2–5 | Longitudinal cohort | Clinical diagnosis | Undifferentiated arthritis | Progression to RA | With the optimal cut-off point of 5 for PD:  Se 0.89  Sp 0.88  LR+ 7.89 | n.a. |
| Filer A 2011(24) | 58 | 38 joints.  All but Hip,  PIP, Jaw, DIP, 1 MTP | Longitudinal cohort | Clinical diagnosis | Undifferentiated arthritis | Reclassification to RA by US (1987 criteria) | US reclassifies 3 out of 29 subjects to RA  PD10 index ≥10  Se 0.79  Sp 0.93  LR+ 11.5  LR- 0.22 | **Intra-reader reliability (kappa)**  GS 0.83  PD 0.87  Erosions 0.93 |
| Tamas S 2013(25) | 100 | 18 joints. Wrist,  MCP 2–5, PIP 2–5 | Cross-sectional cohort | Clinical diagnosis | Subjects with >1 swollen joint | Treatment with DMARDs | US findings is associated with DAS-28 (p = 0.01) and DMARD’s initiation (p = 0.01) | n.a. |
| Navalho M 2013(26) | 45 | 18 joints Wrist, MCP 2–5, PIP 2–5 | Longitudinal cohort | Clinical diagnosis | Subjects with swelling in ≥4 joints | Progression to RA (2010 classification) | AUC (US) = 0.853 | **Intra-reader reliability (kappa)**  Synovitis 0.792 |
| Kawashiri SY 2013(27) | 69 | 22 joints  Wrist, MCP  1–5, PIP 1–5 | Longitudinal cohort | Start of DMARDs within 3 months | Undifferentiated arthritis | Added value of PD for the  performance of 2010 RA classification criteria | PD grade 2 or 3:  PPV 0.94  NPV 0.81 | n.a. |
| Ozgul A  2009(28) | 51 | 40 joints  All but DIP, IP, jaw and hip | Longitudinal cohort | Clinical diagnosis | Undifferentiated arthritis | Development of RA | In the presence of synovitis at US: Kappa value for development of RA = 0.625 | n.a. |
| Nakagomi D 2013(29) | 109 | 38 joints.  All but DIP,  IP, jaw, hip, 1 MTP | Longitudinal cohort | Clinical Diagnosis | Patients with arthralgia | Improvement of the accuracy of the 2010 criteria for RA | **GS ≥ 1**  Se 0.78  Sp 0.79  **GS ≥ 2/PD ≥ 1**  Se 0.56  Sp 0.93 | **Intra-reader reliability (ICC)**  GS 0.94  PD 0.99  **Inter-reader reliability (ICC)**  GS 0.86  PD 0.89 |
| Broll M 2012(30) | 50 | 6 joints.  Wrist and MCP of  dominant hand | Cross-sectional cohort | Clinical Diagnosis | Patients with arthralgia | Diagnostic accuracy | **GS**  Se 0.94  Sp 0.5  **PD**  Se 0.72  Sp 0.94  **Erosions**  Se 0.38  Sp 1 | n.a. |
| Platt AG  2013 (31) | 379 | 16 joints.  MCP/PIP 2–4, MTP 1–2 | Longitudinal cohort | Clinical diagnosis | Patients with arthralgia | Prediction of the development of RA | AUC 0.91 for algorithm without US  AUC 0.91 for algorithm including US | **Inter-reader reliability (kappa)**  GS 0.56  PD 0.64 |
| Rezaei H  2014(32) | 103 | 30 joints  Wrist, MCP,PIP,MTP 2–5 and symptomatic  joints | Longitudinal cohort | Clinical diagnosis | Patients with arthralgia | Added value of US to routine clinical and laboratory examination to diagnose RA | The group of patients with a diagnostic probability from 40 to 60%, dropped from 30 to 10 after US | n.a. |
| Minowa K 2015(33) | 122 | 22 joints. Wrist, MCP, PIP | Longitudinal cohort | Clinical diagnosis | Patients  with arthralgia | Diagnosis of RA | Diagnostic performance for RA in seropositive  group:  PD ≥ 2: OR = 10.48  Diagnostic performance for RA in seronegative group:  PD ≥ 1: OR = 20,  GS = 3: OR = 8.52 | n.a. |
| Zufferey P 2017(34) | 80 | 22 joints  (the same of the DAS28 without thumbs, hips and shoulders) | Retrospective cohort study | Clinical Diagnosis | Seronegative patients with arthralgia | Development of RA | Se 0.57  Sp 0.82  NPV 0.92 | n.a. |
| Ji L 2017(35) | 94 | 22 joints. Wrists, MCP1–5, PIP1–5 | Longitudinal cohort | Clinical Diagnosis | Seronegative patients with undifferentiated arthritis | Added value of US to the 2010 ACR/EULAR classification criteria in the early diagnosis of RA | GS  Se 0.69  Sp 0.86  PD  Se 0.72  Sp 0.87 | n.a. |

***Psoriatic arthritis***

***Flow-chart§***


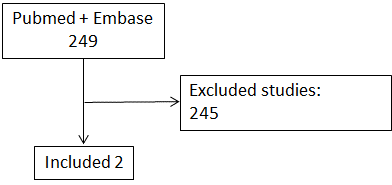


§ 24 studies were retrieved from the previous SLR

***Summary of findings table: US to diagnose PsA***

Summary of findings of studies assessing the performance of US to diagnose PsA. GS: grey scale; PD: power Doppler; Se: sensitivity; Sp: specificity; LR+: positive likelihood ratio; LR-: negative likelihood ratio; MCP: metacarpophalangeal joints; PIP: proximal interphalangeal joints; DIP: distal interphalangeal joints; MTP: metatarsophalangeal joints; PsA: psoriatic arthritis; PsO: psoriasis; RA: rheumatoid arthritis; OA: osteoarthritis; ESR: erythrosedimentation rate; ICC: interclass correlation coefficient; MASEI: Madrid Sonographic Enthesitis Index.

| **Study** | **N** | **Site** | **Study design** | **Comparator/reference standard** | **Population** | **Outcome** | **Diagnostic performance** | **Reliability** |
| --- | --- | --- | --- | --- | --- | --- | --- | --- |
| Freeston 2012(36) | 42 PsA  10 HC | Bilateral lateral epicondyles of the elbow, inferior patellar tendons insertion, Achilles tendons and plantar fascia | Case-control | CASPAR criteria | Recent onset PsA | Clinical diagnosis of PsA | **GS>1 PD>0**  Se 0.08 (0.05,0.11)  Sp 0.88 (0.79,0.94)  LR+ 0.70 (0.34,1.45)  LR- 1.04 (0.95,1.13)  **Erosions**  Se 0.05 (0.02,0.08)  Sp 0.96 (0.89,0.99)  LR+ 1.32 (0.39,4.44)  LR- 0.99 (0.94,1.04)  **Bony spurs**  Se 0.17 (0.13,0.22)  Sp 0.83 (0.73,0.90)  LR+ 1.07 (0.62,1.87)  LR- 0.99 (0.88,1.10)  **Calcifications**  Se 0.02 (0.007,0.04)  Sp 0.97 (0.91,0.99)  LR+ 0.79 (0.16,3.84)  LR- 1.01 (0.97,1.05)  **Intratendineous lesions**  Se 0 (0,0.01)  Sp 1 (0.95,1)  LR- 1 (1,1) | **Intra-reader reliability (kappa)**  GS 0.73(0.6, 0.86)  PD 0.91(0.85,0.86) |
| Gutierrez 2011(37) | 20 PsA  18 RA | MCPs | Case-control | ESSG criteria | peripheral PsA with clinical involvement of at least one MCP joint | Clinical diagnosis of PsA | **Peritendineous effusion**  Se 0.65 (0.54,0.75)  Sp 1 (0.95,1)  LR - 0.34 (0.25,0.46)  **Peritendineous PwD**  Se 0.60 (0.49,0.71)  Sp 0.95 (0.88,0.98)  LR + 12.65 (4.79,33.43)  LR - 0.41 (0.31,0.54)  **Joint space widening**  Se 0.34 (0.24,0.45)  Sp 0 (0,0.04)  LR + 0.34 (0.25,0.46)  **Effusion**  Se 0.07 (0.02,0.15)  Sp 0.81 (0.71,0.89)  LR + 0.40 (0.17,0.99)  LR - 1.13 (1.01,1.27)  **Synovial hypertrophy**  Se 0.26 (0.17,0.37)  Sp 0.18 (0.10,0.28)  LR + 0.33 (0.23,0.47)  LR - 4.05 (2.51,6.52)  **Intraarticular PwD**  Se 0.34 (0.24,0.45)  Sp 0.13 (0.06,0.22)  LR + 0.39 (0.29,0.54)  LR - 4.97 (2.80,8.80) | n.a. |
| Iagnocco 2012(38) | 76 PsA  20 RA | Tendons: common extensor; gluteus; quadriceps; patellar; Achilles tendon; plantar aponeuroses. | Case-control | Clinical diagnosis | PsA | Clinical diagnosis of PsA | **Hypoecogenicity**  Se 0.16 (0.14,0.19)  Sp 0.93 (0.89,0.96)  LR+ 2.68 (1.58,4.52)  LR- 0.88( 0.85,0.927)  **Thickening**  Se 0.06 (0.05,0.08)  Sp 0.91 (0.87,0.94)  LR+ 0.79 (0.49,1.3)  LR- 1.02 (0.97,1.06)  **Calcifications**  Se 0.19 (0.16,0.21)  Sp 0.86 (0.80,0.90)  LR+ 1.37 (0.96,1.95)  LR- 0.94 (0.88,0.99)  **Entesophytes**  Se 0.85 (0.84,0.86)  Sp 0.52 (0.45,0.58)  LR+ 1.79 (1.56,2.05)  LR- 0.27 (0.24,0.32)  **Erosions**  Se 0.06 (0.04,0.07)  Sp 0.97 (0.94,0.99)  LR+ 2.82 (1.15,6.92)  LR- 0.95 (0.93,0.98)  **PD enthesis**  Se 0.04 (0.03,0.06)  Sp 0.98 (0.95,0.99)  LR+ 2.58 (0.94,7.07)  LR- 0.97 (0.95,0.99)  **PD tendon**  Se 0.06 (0.04,0.07)  Sp 0.94 (0.90,0.97)  LR+ 1.12 (0.61,2.04)  LR- 0.99 (0.95,1.03)  **Bursitis**  Se 0.08 (0.06,0.09)  Sp 0.87 (0.82,0.91)  LR+ 0.67 (0.44,1.01)  LR- 1.05 (0.99,1.1)  **Tendon lesion**  Se 0.004 (0.001,0.01)  Sp 0.99 (0.97,1)  LR+ 1.05 (0.12,8.97)  LR- 1 (0.99,1.01) | n.a. |
| Lin 2015(39) | 44 PsA  39 RA  20 HC | fingers (MCP, PIP, DIP, extensor tendons, flexor tendons, soft tissues) | Case-control | CASPAR criteria | PsA with finger involvement | Clinical diagnosis of PsA | **Effusion**  Se 0.61 (0.51,0.69)  Sp 0.32 (0.24,0.41)  LR+ 0.90 (0.75,1.09)  LR- 1.19 (0.85,1.67)  **Synovial** **thickening**  Se 0.55 (0.46,0.64)  Sp 0.36 (0.27,0.45)  LR+ 0.46 (0.38,0.55)  LR- 0.44 (0.34,0.54)  **Erosions**  Se 0.57 (0.48,0.66)  Sp 0.51 (0.42,0.60)  LR+ 1.19 (0.94,1.51)  LR- 0.81 (0.62,1.07)  **Tenosynovitis**  Se 0.57 (0.48,0.66)  Sp 1 (0.97,1)  LR- 0.42 (0.34,0.52)  **Enthesisits**  Se 0.31 (0.23,0.40)  Sp 1 (0.97,1)  LR- 0.68 (0.60,0.77)  **Periosteal** **reaction**  Se 0.17 (0.1,0.24)  Sp 1 (0.97,1)  LR- 0.82 (0.76,0.89)  **Tenoostephyma**  Se 1 (0.81,1)  Sp 0.53 (0.47,0.60)  LR+ 2.16 (1.88,2.49)  **Soft** **tissue** **inflammation**  Se 0.35 (0.27,0.44)  Sp 1 (0.97,1)  LR- 0.64(0.56,0.73) | n.a. |
| Marchesoni 2012 (40) | 30 PsA  30 fibromyalgia | Tendons: common exten, gluteus, quadriceps, patellar, Achilles tendon and plantar fascia. | Case-control | CASPAR criteria | Consecutive patients with PsA | Clinical diagnosis of PsA | **Enthesopathy**  Se 1 (0.88,1)  Sp 0.2 (0.07,0.38)  LR + 1.25 (1.05,1.49)  **Inflammatory lesions**  Se 0.7 (0.5,0.85)  Sp 0.65 (0.40,0.84)  LR + 2 (1.05,3.8)  LR - 0.46 (0.24,0.87)  **Hypoechogenicity**  Se 0.43 (0.25,0.62)  Sp 0.9 (0.68,0.98)  LR + 4.33 (1.09,17.2)  LR - 0.63 (0.44,0.88)  **Erosions**  Se 0.2 (0.07,0.38)  Sp 1 (0.88,1)  LR - 0.8 (0.66,0.95)  **PD**  Se 0.5 (0.31,0.68)  Sp 0.65 (0.40,0.84)  LR + 1.43 (0.71,2.87)  LR - 0.76 (0.47,1.24) | n.a. |
| Melchiorre 2003(41) | 11 PsA  22 RA | temporomandibular joint | case-control | Clinical diagnosis | Active disease with ESR>30 mmh and suspected temporomandibular joint involvement | Clinical diagnosis of PsA | **Meniscal abnormalities**  Se 0.45 (0.16,0.76)  Sp 0.40 (0.20,0.63)  LR + 0.76 (0.36,1.6)  LR – 1.33(0.63,2.79)  **Condyle abnormalities**  Se 0.54 (0.23,0.83)  Sp 0.45 (0.24,0.67)  LR + 1 (0.51,1.94)  LR – 1 (0.45,2.21)  **Effusion**  Se 0.54 (0.23,0.83)  Sp 0.45 (0.24,0.67)  LR + 1 (0.51,1.94)  LR – 1 (0.45,2.21) | **Intra-reader reliability (kappa)**  0.76 |
| Turner 2014(42) | 34 PsA  22 HC | MTP 1-5 | case-control | CASPAR criteria | Patients with a confirmed diagnosis of PsA, based on the CASPAR criteria | Clinical diagnosis of PsA | **GS Synovitis**  Se 0.13 (0.1,0.18)  Sp 1 (0.98,1)  LR- 0.86 (0.82,0.89)  **Erosions**  Se 0.13 (1,0.17)  Sp 1 (0.98,1)  LR- 0.86 (0.82,0.90)  **Effusion**  Se 0.46 (0.41,0.51)  Sp 0.58 (0.51,0.65)  LR+ 1.12 (0.92,1.36)  LR- 0.91 (0.78,1.06)  **PD**  Se 0.16 (0.13,0.20)  Sp 1 (0.98,1)  LR- 0.83 (0.79,0.87) | n.a. |
| Wiell 2007(43) | 15 PsA  5 RA  5 HC | 2-5 MCP, DIP e PIP, 1-5 MTP  [synovitis, bone erosion, bone proliferation, intra-extracapsular PwD (for PIP joints only)] | Case-control | Clinical diagnosis | Patients with PsA and at least one clinically affected finger joint or dactylitis) | Clinical diagnosis of PsA | DIP joints of PsA had more pathological findings than RA; no bone changes (erosions and proliferations) were present in RA. Synovitis was common in both groups. | **Inter-reader reliability (kappa)**  Bone changes 0.52-1  Synovitis 0.78-1 |
| Woodburn 2013(44) | 42 PsA  29 HC with foot or ankle pain | Achilles tendon  [tendon thickness 5.29 mm, retrocalcaneal bursitis, erosions, enthesophytes, PwD] | Case-control | CASPAR criteria | Consecutive patients with PsA | Clinical diagnosis of PsA | Se 0.66 (0.50,0.80)  Sp 0.69 (0.49,0.84)  LR+ 2.15 (1.2,3.85)  LR- 0.48 (0.29,0.79) | n.a. |
| Acquacalda 2015(45) | 34 cutaneous psoriasis | Achilles tendon, plantar fascia insertion, quadriceps insertion, proximal patellar and triceps tendon insertion  [Hypoechogenicity; Thickening; Erosion; Calcification; Power Doppler; Morphological lesions; Structural lesions; All lesions] | Longitudinal cohort (6 months) | CASPAR criteria | Cutaneous psoriasis starting the first systemic treatment | Clinical diagnosis of PsA | Se 0.52 (0.43,0.62)  Sp 0.53 (0.46,0.60)  LR+ 1.14 (0.92,1.43)  LR- 0.88 (0.70,1.10) | n.a. |
| Eder 2014(46) | 50 PsA  66 cutaneous psoriasis  60 HC | insertions of the quadriceps and patellar tendons, Achilles tendon and plantar fascia insertions, triceps tendon insertion | Case-control | CASPAR criteria | Consecutive PsA | Clinical diagnosis of PsA | Se 0.30 (0.16,0.46)  Sp 0.89 (0.79,0.95)  LR+ 2.83 (1.21,6.59)  LR- 0.78 (0.63,0.97) | **Intra-reader reliability (ICC)**  MASEI 0.8 |
| Ciancio 2014(47) | 150 PsA  172 SpA  95 HC | lateral surface of the fifth MTP  [bursitis] | Case-control | CASPAR criteria | Consecutive PsA | Clinical diagnosis of PsA | Se 0.10 (0.05,0.16)  Sp 1.0 (0.96,1.0)  LR- 0.90 (0.85,0.95) | **Intra-reader reliability (kappa)**  Bursitis 0.96  **Inter-reader reliability (kappa)**  Bursitis 0.87 |
| Falcao 2013(48) | 66 PsA  RA and HC gender-matched | Achilles tendon bursae | Case-control | modified New York criteria, CASPAR, ASAS, ESSG criteria | Consecutive PsA | Clinical diagnosis of PsA | Se 0.67 (0.58,0.75)  Sp 0.59 (0.49,0.69)  LR+ 1.68 (1.27,2.21)  LR- 0.54 (0.40,0.73) | **Intra-reader reliability (kappa)**  0.82 |
| Ezzat 2013(49) | 42 SpA including 10 PsA  20 HC | Patellar tendon origin and insertion, Achilles tendon, plantar aponeurosis | Case-control | ASAS criteria | ASAS criteria | Clinical diagnosis of PsA | Se 0.64 (0.59,0.68)  Sp 1.0 (0.83, 1.0)  LR- 0.35 (0.31-.040) | n.a. |
| Bandinelli 2013(50) | 92 PsA  40 HC | quadriceps, patellar, achilles tendons and plantar fascia | Case-control | CASPAR criteria | GUESS Early PsA | Clinical diagnosis of PsA | **PsA vs HC**  Thickness: 94.5% vs 0%  Enthesophytes: 82.6% vs 5%  Bursitis: 21.7% vs 0%  Erosions: 10.8% vs 0%  PwD: 40.2% vs 0% | **Intra-reader reliability (ICC)**  GUESS 0.99 (0.98,1)  PD 0.97 (0.9,1) |
| Aydin 2013(51) | 58 PsA  42 Pso  23 HC | GUESS score modified  Hypoechogenicity; Thickening; Erosion; Calcification; PwD; entesophytosis, bursitis | Case-control | CASPAR criteria | Cutaneous psoriasis including PsA | Clinical diagnosis of PsA | **PsA vs HC (% of joints)**  Hypoechogenicity 35.9% vs 12.6%  Thickening 43.1% vs 30%  PD 2.1% vs 0 %  Calcifications 9.8% vs 9.6%  Bursal enlargement 13.3% vs 1.3%  Bursal PD 2.8% vs 0%  Entesophytes 51.2% vs 39.1%  Erosions 5% vs 0.4%  **PsA vs PsO (% of joints)**  Hypoechogenicity 35.9% vs 25%  Thickening 43.1% vs 38.6%  PD 2.1% vs 0.4%  Calcifications 9.8% vs 4.1%  Bursal enlargement 13.3% vs Pso 8.3%  Bursal PD 2.8% vs 0.7%  Entesophytes 51.2% vs 42.9%  Erosions 5% vs 2.9% | **Inter-reader reliability (ICC)**  GS inflammation 0.91-0.93  PD inflammation 0.74-0.95  Chronicity scores 0.89-0.93  US score 0.92-0.95 |
| Aydin 2013(52) | 18 Cutaneous psoriasis including PsA  12 HC | Bilateral hand nails | Case-control | Clinical diagnosis of psoriasis | Cutaneous psoriasis with nail disease | Clinical diagnosis of PsA | Se 0.66 (0.40,0.86)  Sp 1.0 (0.73,1.0)  LR- 0.33 (0.17,0.64) | n.a. |
| Aydin 2012(53) | 86 Cutaneous psoriasis including PsA  20 HC | Bilateral hand nails | Case-control | Clinical diagnosis of nail involvement | Cutaneous psoriasis including PsA | Clinical diagnosis of PsA | Se 0.50 (0.39,0.60)  Sp 0.90 (0.68,0.98)  LR+ 5.00 (1.32,18.93)  LR- 0.56 (0.43,0.72) | **Intra-reader reliability (kappa)**  0.58 |
| De Simone 2011(54) | 100 Cutaneous psoriasis  Skin psoriasis sex- and age-matched, same inclusion criteria but no pain | MCP, PIP, DIP, MTP | Case-control | Expert opinion to diagnose PsA | Pso with symptoms: >18 years, diagnosis of psoriasis for at least a year, pain involving any finger and/or toe  joint for >3 months. | Clinical diagnosis of PsA | **Overall**  Se 1 (0.90,1.0)  Sp 1 (0.79, 1.0)  **PD**  Se 0.80 (0.63,0.91)  Sp 0.62 (0.35,0.84)  LR+ 2.15 (1.12,4.31)  LR- 0.31 (0.14,0.67) | n.a. |
| Farouk 2010(55) | 30 cutaneous psoriasis  30 PsA | Achilles tendon | Case-control | CASPAR criteria | Skin psoriasis without known arthropathy | Clinical diagnosis of PsA | Se 0.41 (0.24,0.59)  Sp 0.61 (0.40,0.79)  LR+ 1.07 (0.57,2.01)  LR- 0.96 (0.63,1.45) | n.a. |
| Falsetti 2003(56) | 125 PsA  Erosive OA, nodular OA, RA, HC | Achilles tendon and enthesis; retrocalcaneal bursae; plantar fascia and enthesis; subcalcaneal fat pad; cortical bone of posterior and inferior aspects of heel | Case-control | Moll and Wright criteria for PsA | PsA | Clinical diagnosis of PsA | Posteroinferior enthesophytosis, subcalcanear panniculitis more frequent than RA and HC  Achilles tendon enthesitis more frequent than erosive OA, RA, HC  Deep retrocalcanear bursitis, posterior erosions more frequent than nodal OA, RA, HC  Inferior enthesophytosis more frequent than HC  Inferior erosion not significantly different from controls  Plantar fasciitis more frequent than all controls | n.a. |
| Falsetti 2002(57) | SpA including 41 PsA  100 RA, 100 OA, 100 with Painful Shoulder, 50 HC | Bilateral shoulders | Case-control | Moll and Wright criteria for PsA | Patients with PsA and shoulder pain | Clinical diagnosis of PsA | ***Enthesitis of the proximal insertion of the deltoid***  Se 0.21 (0.10,0.37)  Sp 1.0 (0.92,1.0)  LR – 0.78(0.66,0.92) | n.a. |
| Fourniè 2006(58) | 20 PsA  21 RA | Dorsal, volar, and lateral aspects of the finger. | Case-control | Fourniè criteria for PsA | PsA with symptoms in one or more fingers | Clinical diagnosis of PsA | **Synovitis**  Se 0.76(0.54,0.90)  Sp 0 (0,0.13)  LR+ 0.76(0.61,0.95)  **Tenosynovitis**  Se 0.16 (0.04,0.36)  Sp 0.56 (0.34,0.75)  LR+ 0.36 (0.13,0.99)  LR – 1.50 (1.02,2.21)  **Enthesitis**  Se 0.51 (0.32,0.72)  Sp 0.40 (0.21,0.61)  LR+ 0.87 (0.53,1.42)  LR – 1.20 (0.64,2.25) | n.a. |
| Mendonça 2014(59) | 28 PsA  7 (HC+OA) | Ungueal beds | Case-control | CASPAR criteria | Patients with a confirmed diagnosis of PsA, based on the CASPAR criteria | Clinical diagnosis of PsA | Spectral Doppler may help confirm quantitatively ungueal inflammatory activity in PsA patients | n.a. |
| Zabotti A 2016(60) | 26 early PsA  34 early RA | 2 most clinically involved joints; dactylitis excluded. | Case-control | CASPAR criteria | Patients with symptom duration <12 months and prevalent involvement of the hands | Clinical diagnosis of PsA | **Fingers as statistical units**  **Soft tissue oedema**  Se 0.42 (0.23,0.63)  Sp 0.97 (0.84,0.99)  LR+ 14.38 (1.98,104.44)  LR- 0.59 (0.43, 0.83)  **Synovitis (GS)**  Se 0.56 <80.45,0.73)  Sp 0.09 (0.03,0.18)  LR+ 0.65 (0.52,0.83)  LR- 4.58 (1.99,10.52)  **Synovitis (PD)**  Se 0.81 (0.62,0.92)  Sp 0.05 (0.01,0.13)  LR+ 0.85 (0.71,1.02)  LR- 4 (1.07,14.93)  **Peritendinitis**  Se 0.59 (0.36,0.79)  Sp 0.97 (0.86,0.99)  LR+ 23.05 (3.23,164.54)  LR- 0.42 (0.25,0.70)  **Central slip enthesitis**  Se 0.25 (0.11,0.44)  Sp 1 (0.88,1)  LR- 0.75 (0.61,0.93)  **Erosions**  Se 0.04(0.005,0.13)  Sp 0.87 (0.76,0.94)  LR+ 0.29 (0.07,1.29)  LR- 1.11 (1,1.23)  **Flexor tenoynovitis (PD)**  Se 0.53 (0.33,0.73)  Sp 0.35 (0.19,0.53)  LR+ 0.83 (0.54,1.28)  LR- 1.31 (0.71,2.42) | n.a. |
| Groves C 2017(61) | 11 PsA  9 RA | Common extensor, common flexor, tricipital entheses at the elbow | Case-control | CASPAR criteria | Patients with a physician diagnosis of PsA  and RA that complained of pain  at the elbow. | Clinical diagnosis of PsA | PsA mean inflammation and damage scores of 1.82 and  0.55. RA mean inflammation and damage scores of 1.57 and 1.50 (n.s.) | n.a. |

***Polymyalgia Rheumatica***

***Flow-chart§***


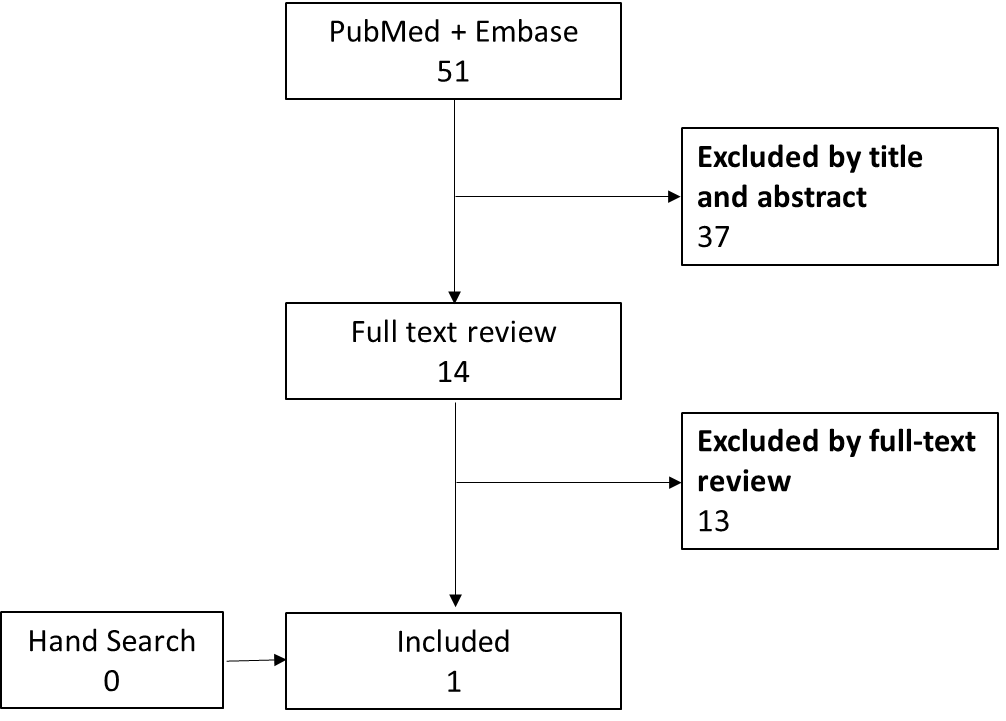


§ 10 studies were retrieved from the previous SLR

***Summary of findings table: US to diagnose PMR***

Summary of findings of studies assessing the performance of US to diagnose PMR. US: ultrasonography; RA: rheumatoid arthritis; SpA: spondyloarthritis; LHBT: long head of the biceps tendon; LH; long head of the biceps; GH: gleno-humeral; SAD: subacromiodeltoid; Se: sensitivity; Sp: specificity; SAD: subacromiondeltoid bursa; BB: brachialis biceps.

| **Study** | **N** | **Site** | **Study design** | **Comparator/reference standard** | **Population** | **Outcome** | **Diagnostic performance** | **Reliability** |
| --- | --- | --- | --- | --- | --- | --- | --- | --- |
| Dasgupta B 2012(62) | 125 PMP  169 controls | Shoulders (LHBY, SAD bursa, GH) and hips (hip synovitis) | Longitudinal cohort | Clinical diagnosis | New onset bilateral shoulder pain | Diagnosis of PMR | ***SAD bursitis at least by one side***  Se 0.56 (0.47,0.65)  Sp 0.65 (0.58,0.72)  LR+ 1.6 (1.2,2.1)  LR- 0.67 (0.53,0.85)  *Vs RA*  Se 0.56 (0.47,0.65)  Sp 0.72 (0.57,0.83)  LR+ 2 (1.2,3.2)  LR- 0.6(0.46,0.80)  *Vs painful shoulder*  Se 0.56 (0.47,0.65)  Sp 0.70 (0.55,0.81)  LR+ 1.9 (1.2,2.9)  LR- 0.61 (0.46,0.80)  ***SAD bursitis, bilateral***  Se 0.32 (0.24,0.41)  Sp 0.88 (0.81,0.92)  LR+ 2.6 (1.6,4.2)  LR- 0.38 (0.15,0.97)  *Vs RA*  Se 0.32 (0.24,0.41)  Sp 0.78 (0.64,0.88)  LR+ 1.5 (0.8,2.7)  LR- 0.87 (0.64,0.88)  ***Trochanteric bursitis at least by one side***  Se 0.21 (0.15, 0.30)  Sp 0.91 (0.84,0.95)  LR+ 2.3 (1.2,4.5)  LR- 0.87 (0.78,0.97  ***LHB tenosynovitis at least by one side***  Se 0.66 (0.57,0.74)  Sp 0.54 (0.46,0.61)  LR+ 1.4 (1.2,1.8)  LR- 0.63 (0.47,0.85)  *Vs RA* Se 0.66 (0.57,0.74)  Sp 0.44 (0.31,0.59)  LR+ 1.2 (0.89,1.6)  LR- 0.76 (0.51,1.2)  *Vs painful shoulder*  Se 0.66 (0.57,0.74)  Sp 0.60 (0.45,0.72)  LR+ 1.6 (1.1., 2.4)  LR- 0.57 (0.40,0.80)  ***LHB tenosynovitis, bilateral***  *Vs RA*  Se 0.37 (0.29,0.46)  Sp 0.62 (0.48,0.75)  LR+ 0.99 (0.63,1.5)  LR- 1 (0.77,1.3)  ***GH synovitis at least by one side***  Se 0.39 (0.30,0.48)  Sp 0.71 (0.64,0.78)  LR+ 1.3 (0.96,1.9)  LR- 0.86 (0.72,1)  *Vs RA*  Se0.39 (0.30,0.48)  Sp 0.63 (0.49,0.75)  LR+ 1.4 (0.99,1.9)  LR- 0.66 (0.39,1.1)  *Vs painful shoulder*  Se 0.39 (0.3,0.48)  Sp0.76 (062,0.86)  LR+1.6 (0.92,2.8)  LR-0.81 (0.65,1)  ***GH synovitis, bilateral***  Se 0.26 (0.19,0.35)  Sp 0.83 (0.76,0.88)  LR+ 1.5 (0.97,2.4)  LR-0.89 (0.78,1)  *Vs RA*  Se 0.26 (0.19,0.35)  Sp0.70 (0.55, 0.81)  LR+ 0.86 (0.50,1.5)  LR- 1.1 (0.85,1.3)  ***Hip synovitis at least by one side***  Se 0.26 (0.19,0.36)  Sp 0.81 (0.73,0.87)  LR+ 1.4 (0.87,2.3)  LR- 0.90 (0.78,1)  ***Hip synovitis, bilateral***  Se 0.18 (0.12,0.26)  Sp 0.92 (0.85,0.95)  LR+ 2.1 (1,4.3)  LR- (0.90 (0.81,1)  ***Bilateral shoulder region inflammation***  Se 0.59 (0.50,0.68)  Sp 0.57 (0.49,0.65)  LR+ 1.4 (1.1,1.7)  LR- 0.71 (0.55, 0.92)  *Vs RA*  Se 0.59 (0.5,0.68)  Sp 0.35 (0.23,0.49)  LR+ 0.91 (0.70,1.2)  LR- 1.2 (0.75,1.8)  *Vs painful shoulder*  Se 0.59 (0.50,0.68)  Sp 0.74 (0.61,0.85)  LR+ 2.3 (1.4,3.9)  LR- 0.55 (0.42,0.72)  ***Hip region inflammation***  *Vs RA*  Se 0.38 (0.30,0.47)  Sp 0.70 (0.55,0.81)  LR+ 1.3 (0.77,2.1)  LR- 0.89 (0.70,1.1)  *Vs painful shoulder*  Se 0.38 (0.30, 0.47)  Sp 0.83 (0.71,0.91)  LR+ 2.3 (1.2,4.4)  LR-0.74 (0.61,0.90)  ***One shoulder and one hip region inflammation***  Se 0.33 (0.26,0.42)  Sp 0.84 (0.77,0.89)  LR+ 2.1 (1.3,3.2)  LR- 0.80 (0.69,0.92) | n.a. |
| Ruta S 2012(63) | 30 PMR  30 RA  30 controls | Shoulders  (LHBT, SAD bursa, GH) | Case-control | Clinical diagnosis | Patients with PMR and RA and shoulder pain  Controls with unilateral shoulder pain | Diagnosis established by the treating physician | ***SAD bursitis at least by one side***  *Vs RA*  Se 0.73 (0.56,0.86)  Sp 0.67 (0.49,0.81)  LR+ 2.2 (1.3,3.8)  LR- 0.40 (0.21,0.76)  *Vs painful shoulder*  Se 0.55 (0.43,0.67)  Sp 0.75 (0.63,0.84)  LR+ 2.2 (1.3,3.6)  LR- 0.60 (0.44,0.82)  ***LHB tenosynovitis at least by one side***  *Vs RA* Se 0.63 (0.46,0.78)  Sp 0.57 (0.39,0.73)  LR+ 1.5 (0.89,2.4)  LR- 0.65 (0.37,1.1)  *Vs painful shoulder*  Se 0.47 (0.35,0.39)  Sp 0.80 (0.68,0.88)  LR+ 2.3 (1.3,4.1)  LR- 0.67 (0.51,0.87)  ***LHB tenosynovitis, bilateral***  *Vs RA*  Se 0.30 (0.17,0.48)  Sp 0.98 (0.86,1)  LR+ 19 (1.2,310)  LR- 0.71 (0.56,0.90)  ***GH synovitis at least by one side***  *Vs RA*  Se 0.20 (0.10,0.37)  Sp 0.57 (0.39,0.73)  LR+ 0.46 (0.20,1.1)  LR- 1.4 (0.99,2)  *Vs painful shoulder*  Se 0.12 (0.058,0.22)  Sp 0.93 (0.84,0.97)  LR+ 1.8 (0.54,5.7)  LR-0.95 (0.84,1.06)  ***GH synovitis, bilateral***  *Vs RA*  Se 0.03 (0.059,0.17)  Sp 0.90 (0.74,0.97)  LR+ 0.37 (0.037,3)  LR- 1.1 (0.94,1.2) | n.a. |
| Falsetti P 2011 (64) | 61 | Shoulders (SAD bursa, GH) and hips (hip synovitis) | Corss-sectional cohort | Clinical diagnosis | Elderly patients with polymyalgic symptoms | Diagnosis established by the treating physician | ***SAD bursitis at least by one side***  Se 0.79 (0.62,0.90)  Sp 0.59 (0.42,0.74)  LR+ 2 (1.2,3.1)  LR- 0.35 (0.16,0.75)  ***SAD bursitis, bilateral***  Se 0.69 (0.51,0.83)  Sp 0.78 (0.61,0.89)  LR+ 3.2 (1.6,6.3)  LR- 0.40 (0.22,0.70)  *Vs RA*  Se 0.37 (0.22,0.55)  Sp 0.78 (0.64,0.88)  LR+ 11 (1.5,80)  LR- 0.66 (0.83,0.99)  ***GH synovitis at least by one side***  Se 0.66 (0.47,0.80)  Sp 0.47 (0.31,0.64)  LR+ 1.2 (0.81,1.9)  LR-0.74 (0.4,1.4)  ***GH synovitis, bilateral***  Se 0.48 (0.31,0.66)  Sp 0.66 (0.48,0.80)  LR+ 1.4 (0.76,2.6)  LR-0.79 (0.51,1.2)  ***Hip synovitis at least by one side***  Se 0.24 (0.12,0.42)  Sp 0.88 (0.72,0.95)  LR+ 1.9 (0.63,5.9)  LR - 0.87 (0.68, 1.1) | n.a. |
| Cantini F 2001(65) | 57 PMR  114 controls | Shoulders (LHBT, SAD bursa, GH) | Case-control | Clinical diagnosis | Consecutive patients with untreated PMR  Next 2 consecutive patients with shoulder pain and stiffness seen after the case | Diagnosis established by the treating physician | ***SAD bursitis at least by one side***  Se 0.96 (0.88,0.99)  Sp 0.78 (0.7,0.85)  LR+ 4.4. (3.1,6.2)  LR- 0.04 (0.01,0.18)  ***SAD bursitis, bilateral***  Se 0.93 (0.83,0.97)  Sp 0.99 (0.95,1)  LR+ 106 (15,747)  LR- 0.07 (0.028,0.18)  ***LHB tenosynovitis at least by one side***  Se 0.81 (0.70,0.89)  Sp 0.47 (0.38,0.57)  LR+ 1.5 (1.2,1.9)  LR- 0.41 (0.23,0.72)  ***GH synovitis at least by one side***  Se 0.77 (0.65,0.86)  Sp 0.42 (0.33,0.51)  LR+ 1.3 (1.1,1.6)  LR- 0.54 (0.32,0.92) | n.a. |
| Frediani B 2002(66) | 50 PMR  50 SpA  50 RA | Shoulders (LHBT, SAD bursa, GH) and hips (hip synovitis) | Case-control | Clinical diagnosis | Conscecutive patientes with PMR, SpA and RA | Diagnosis established by the treating physician | ***SAD bursitis at least by one side***  Se 0.70 (0.56,0.81)  Sp 0.61 (0.51,0.70)  LR+ 1.8 (1.3,2.4)  LR- 0.49 (0.31,0.77)  ***SAD bursitis, bilateral***  Se 0.54 (0.40,0.67)  Sp 0.68 (0.58,0.76)  LR+ 1.7 (1.1, 2.5)  LR- 0.68 (0.49,0.94)  ***LHB tenosynovitis at least by one side***  Se 0.68 (0.54,0.79)  Sp 0.59 (0.49,0.68)  LR+ 1.7 (1.2,1.8)  LR- 0.63 (0.47,0.85)  ***GH synovitis at least by one side***  Se 0.66 (0.52,0.78)  Sp 0.65 (0.55,0.73)  LR+ 1.9 (1.4,2.6)  LR- 0.52 (0.35,0.79)  ***GH synovitis, bilateral***  Se 0.52 (0.39,0.65)  Sp 0.78 (0.69, 0.85)  LR+2.5 (1.5,3.7)  LR- 0.62 (0.45,0.84)  ***Hip synovitis at least by one side***  Se 0.40 (0.28,0.54)  Sp 0.81 (0.72,0.87)  LR+ 2.1 (1.2,3.6)  LR- 0.74 (0.58,0.95)  ***Hip synovitis, bilateral***  Se 0.32 (0.21,0.46)  Sp 0.83 (0.75,0.89)  LR+ 1.88 (1,3.4)  LR- 0.82 (0.66,1) | n.a. |
| Cantini F 2005(67) | 20 PMR  40 controls | Hips  (iliopsoas bursa, trochanteric bursa, hip synovitis) | Case-control | Clinical diagnosis | Consecutive patients with PMR | Diagnosis established by the treating physician | ***Iliopsoas bursitis***  Se 0.30 (0.15,0.50)  Sp 0.90 (0.77, 0.96)  LR+ 3 (0.95,9.4)  LR- 0.78 (0.57,1.1.)  *Vs RA*  Se 0.20 (0.081,0.42)  Sp 0.95 (0.84,0.66)  LR+ 4 (0.80,20)  LR- 0.84 (0.70,1.1)  ***Trochanteric bursitis at least by one side***  Se 098 (0.81,1)  Sp 0.70 (0.54,0.81)  LR+ 3.2 (2,5.1)  LR- 0.32 (0.14,0.76)  ***Hip synovitis at least by one side***  Se 0.45 (0.25,0.66)  Sp 0.55 (0.40,0.69)  LR+ 1 (0.55,1.8)  LR- 1 (0.62,1.6) | n.a. |
| Coari G 1999(68) | 32 PMR  90 RA  122 periarticular disorders  108 controls | Shoulders  (LHBT, GH and SAD bursa) | Case-control | Clinical diagnosis | n.s. | Diagnosis established by the treating physician | ***SAD bursitis at least by one side***  *Vs RA*  Se 0.09 (0.03,0.24)  Sp 0.90 (0.84,0.94)  LR+ 0.95 (0.29,3.2)  LR- 1.01 (0.89,1.1)  ***LHB tenosynovitis at least by one side***  *Vs RA* Se 0.16 (0.07,0.32)  Sp 0.48 (0.38,0.58)  LR+ 0.30 (0.13,0.69)  LR- 1.8 (1.4,2.3)  *Vs painful shoulder*  Se 0.16 (0.069,0.32)  Sp 0.45 (0.37,0.54)  LR+ 0.28 (0.13,0.65)  LR-1.9 (1.5,2.4)  ***GH synovitis at least by one side***  *Vs RA*  Se 0.66 (0.48, 0.80)  Sp (0.52 (0.42,0.62)  LR+ 1.4 (0.99,1.9)  LR- 0.66 (0.39,1.1)  *VS painful shoulder*  Se 0.66 (0.48,0.80)  Sp 0.77 (0.69,0.84)  LR+ 2.9 (1.9,4.3)  LR-0.45 (0.27,0.73) | n.a. |
| Lange U 2000(69) | 21 PMR  29 controls | Shoulders  (LHBT and GH) | Cross-sectional cohort | Clinical diagnosis | Consecutive patients with shoulder pain | Diagnosis established by the treating physician | ***LHB tenosynovitis at least by one side***  *Vs RA* Se 0.14 (0.05,0.33)  Sp 0.59 (0.41,0.74)  LR+ 0.74 (0.35,1.6)  LR- 1.3 (0.80,2)  ***GH synovitis at least by one side***  *Vs RA*  S0.41 (0.23,0.61)  SP 0.34 (0.20,0.53)  LR+ (0.62 (0.35,1.1)  LR- 1.7 (0.93,3.2) | n.a. |
| Lange U 1998 | 13 PMR  19 controls | Shoulders  (LHBT and GH) | Cross-sectional cohort | Clinical diagnosis | Consecutive patients with shoulder pain | Diagnosis established by the treating physician |  | n.a. |
| Macchioni P 2014 (70) | 136 PMR  149 controls | Shoulders and hips | Longitudinal cohort | Clinical diagnosis | Consecutive patients with shoulder pain | Performance of the 2012 classification criteria for PMR | ***Bilateral shoulder region inflammation***  Se 0.45 (0.39,0.51)  Sp 0.60 (0.47,0.72)  LR+ 1.1 ((0.79,1.6)  LR-0.91 (0.71,1.2)  *Vs RA*  Se 0.56 (0.49,0.63)  Sp 0.74 (0.60,0.85)  LR+ 2.2 (1.3,3.7)  LR- 0.60 (0.47,0.76)  ***One shoulder and one hip region inflammation***  Se 0.34 (0.27,4.1)  Sp 0.77 (0.68,0.85)  LR+ 1.5 (0.97,2.3)  LR- 0.86 (0.74,1) | n.a. |
| Weigand S 2014(71) | 54 | SAD bursa  long head BB hip and shoulder joints | Cross-sectional cohort | Classification Criteria (ACR/EULAR) 2012 | Consecutive patients with onset of polymyalgia | Improvement of Classification Criteria (ACR/EULAR) 2012 adding US of SAD bursa, tenosynovitis of long head BB and synovitis of hips and shoulder joints | Decreased Se with US 0.85🡪0.81 | n.a. |

***CPPD***

***Flow-chart§***


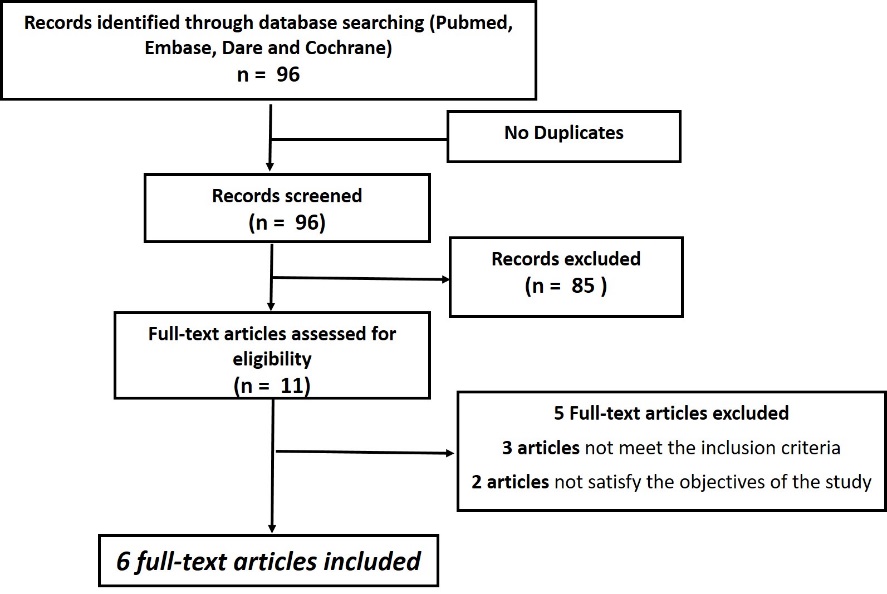


§ 12 studies included in the previous review

***Summary of findings table: US to diagnose CPPD elementary lesions***

Summary of findings of studies assessing the performance of US to diagnose CPPD elementary lesions. Estimates of diagnostic performance are presented as point estimate and 95% confidence intervals, unless specified. Calcium pyrophosphate (CPP); Hyaline Cartilage (HC), Fibrocartilage (FC), Synovial Fluid Analysis (SF), Microscopic Analysis (MA), Triangular Fibrocartilage Complex (TFCC); Se: sensitivity; Sp: specificity; PPV: positive predictive value; NPV: negative predictive value.

| **Study** | **N** | **Site** | **Study design** | **Comparator/reference standard** | **Outcome** | | |
| --- | --- | --- | --- | --- | --- | --- | --- |
|  |  |  |  |  |  | **Diagnostic performance** | **Reliability (inter-reader)** |
| Coari 1995 (72) | 28 Cases  46 Controls | Knee HC | Case-Control | Conventional radiography | CPP Deposits Identification | Se 0.80 (0.66,0.90)  Sp 0.00 (0.00,0.46)  PPV 0.89 (0.78,0.96)  NPV 0.87 (0.85,0.88)  LR+ 0,8  LR- n.a. | n.a. |
| Coari 1995(72) | 28 Cases  46 Controls | Knee FC | Case-Control | Conventional radiography | US Identification of CPP deposits | Se 0.07 (0.02,0.20)  Sp 1 (0.78,1.00)  PPV 1.00  NPV 0.28 (0.26,0.30)  LR+ n.a.  LR- 0.927 | n.a. |
| Foldes 2002 (73) | 21 Cases  19 controls | Knee HC | Case-Control | Conventional radiography | US Identification of CPP deposits | Se 0.89 (0.74,0.97)  Sp 0.91 (0.78,0.97)  PPV 0.89 (0.76,0.95)  NPV 0.91 (0.80,0.96)  LR+ 9,78  LR- 0.12 | n.a. |
| Falsetti 2004(74) | 57 Cases  100 controls | Achilles Tendon | Case-Control | McCarty Criteria | US Identification of CPP deposits | Se 0.58 (0.44,0.71)  Sp 1.00 (0.96,1.00)  PPV n.a.  NPV 0.42 (0.31,0.57)  LR+ n.a.  LR- 0.421 | n.a. |
| Falsetti 2004(74) | 57 Cases  100 controls | Plantar Fascia | Case-Control | McCarty criteria | Us Identification of CPP deposits | Se 0.16 (0.07,0.28)  Sp 0.99 (0.95,1.00)  PPV 0.36 (0.29,0.44)  NPV 0.9 (0.54,0.98)  LR+ 15,79  LR- 0.851 | n.a. |
| Filippucci 2009(75) | 80 Cases 52 controls | Knee HC | Case-Control | SFA | US Identification of CPP deposits | Se 0.69 (0.54,0.81)  Sp 0.98 (0.92,1.00)  PPV 0.94 (0.80,0.98)  NPV 0.84 (0.78,0.89)  LR+ 28,875  LR- 0.32 | n.a. |
| Ellabban 2012(76) | 38 Cases  22 controls | Achilles Tendon | Case-control | McCarty Criteria | US Identification of CPP deposits | Se 0.58 (0.41,0.74)  Sp 1.00 (0.85,1.00)  PPV 1.00  NPV 0.56 (0.49,0.67)  LR+ n.a.  LR- 0.421 | n.a. |
| Ellabban 2012 (76) | 38 Cases  22 controls | Plantar Fascia | Case-control | McCarty criteria | US Identification of CPP deposits | Se 0.16 (0.06,0.31)  Sp 1.00 (0.85,1.00)  PPV 1.00  NPV 0.41 (0.37,0.44)  LR+ n.a.  LR- 0.842 | n.a. |
| Filippou 2012(77) | 6 patients | Knee FC | Cohort Study | MA | US Identification of CPP deposits | Se 0.56 (0.21,0.86)  Sp 0.50 (0.01,0.99)  PPV 0.83 (0.53,0.96)  NPV 0.20 (0.5,0.54)  LR+ 1.11  LR- 0.889 | n.a. |
| Barskova 2013(78) | 25 patients | Knee HC | Cohort Study | SFA | US Identification of CPP deposits | Se 1.00 (0.86,1.00) | n.a. |
| Guiterrez 2014(79) | 74 Cases  83 controls | Knee HC | Case-control | SFA | US Identification of CPP deposits | Se 0.59 (0.47,0.71)  Sp 1.00 (0.95,1.00)  PPV 1.00  NPV 0.73 (0.67,0.78)  LR+ n.a.  LR- 0.405 | 0.72 |
| Guiterrez 2014(79) | 74 Cases  83 controls | Knee FC | Case-control | SFA | US Identification of CPP deposits | Se 0.91 (0.81,0.96)  Sp 1.00 (0.95,1.00)  PPV 1.00  NPV 0.92 (0.91,0.98)  LR+ n.a.  LR- 0.095 | 0.68 |
| Filippou 2014(80) | 42 patients | Knee HC | Cohort Study | MA | US Identification of CPP deposits | Se 0.50 (0.19,0.81)  Sp 1.00(0.74,1.00) | n.a. |
| Filippou 2014(80) | 42 patients | Knee FC | Cohort Study | MA | US Identification of CPP deposits | Se 0.96 (0.80,1.00)  Sp 0.88 (0.62,0.98) | n.a. |
| Juge 2014(81) | 16 Cases  16 Controls | Knee FC | Case-control | SFA | US Identification of CPP deposits | Se 0.94 (0.70,1.00)  Sp 0.88 (0.62,0.98) | 0.81 |
| Juge 2014(81) | 16 Cases  16 Controls | Knee HC | Case-control | SFA | US Identification of CPP deposits | Se 0.75 (0.48,0.93)  Sp 0.94 (0.70,1.00) | 0.81 |
| Ottaviani 2015(82) | 51 patients | Knee HC | Cohort Study | SFA | US Identification of CPP deposits | Se 0.76 (0.55,0.91)  Sp 0.96 (0.80,1.00)  PPV 0.95 (0.73,0.99)  NPV:0.81 (0.67,0.89)  LR+ 19.76  LR- 0.25 | 0.81 |
| Ottaviani 2015(82) | 51 patients | Knee FC | Cohort Study | SFA | US Identification of CPP deposits | Se 0.96 (0.80,1.00)  Sp 0.92 (0.75,0.99)  PPV: 0.92 (0.76,0.98)  NPV: 0.96 (0.78,0.99)  LR + 1.48  LR- 0.043 | 0.81 |
| Di Matteo 2017(83) | 36 Cases  48 Controls | TFCC | Case Control | Mc Carty criteria | US Identification of CPP deposits | Se 0.78 (0.71,0,84)  Sp 0,91 (0.85,0.94)  PPV 0.86 (0.77,0.92)  NPV 0.84 (0.78,0.89)  LR+ 8.296  LR- 0.245 | n.a. |
| Forien 2017 (84) | 32 cases  26 controls | TFCC | Case Control | SFA | US identification of CPP deposits | Se 0.81  Sp 0.85  PPV 0.87  NPV 0.79  LR+ 5.28  LR- 0.22 | n.a. |
| Forien 2017(84) | 32 cases  26 controls | Radio-carpal joint | Case Control | SFA | US identification of CPP deposits | Se 0.5  Sp 0.92  PPV 0.89  NPV 0.60  LR+ 6.5  LR- 0.542 | n.a. |

***Summary of findings table: US to diagnose CPPD***

***S***ummary of findings of studies assessing the performance of US to diagnose CPPD. Estimates of diagnostic performance are presented as point estimate and 95% confidence intervals, unless specified. SFA: synovial fluid analysis; Se: sensitivity; Sp: specificity, PPV: positive predictive value; NPV: negative predictive value; US: ultrasonography.

| **Study** | **N** | **Site** | **Study design** | **Comparator/reference standard** | **Population** | **Outcome** | | |
| --- | --- | --- | --- | --- | --- | --- | --- | --- |
|  |  |  |  |  |  |  | **Diagnostic performance** | **Reliability (inter-reader)** |
| Filippou 2007(85) | 14 Cases 29 controls | Knee | Case-Control | SFA | Two groups of patients. In the first, patients with knee effusion and  US features of CPPD. In the second, the patients had knee effusion  without US signs of CPPD. | US Diagnosis of CPPD | Se 0.87 (0.60,0.98)  0.97 (0.83,1.00)  PPV 0.93 (0.65,0.99)  NPV 0.93 (0.79,0.98)  LR+ 24,26  LR- 0.138 | n.a. |
| Ellabban 2012(76) | 60 patients | Knee | Case-control | SFA | A cohort of patients with knee effusion was divided in two groups.  In the first, the patients were positive for CPP crystals according to SFA. In the second, the patients were negative for CPP crystals at  SFA. | US Diagnosis of CPPD | Se 0.84 (0.69,0.94)  Sp 1.00 (0.79,1.00)  PPV 1.00  NPV 0.78 (0.64,0.88)  LR+ NA  LR-0.158 | n.a. |
| Catay 2013(86) | 39 patients | Knee | Cohort study | SFA | Consecutive patients > 50 years with knee effusion on clinical  examination | US Diagnosis of CPPD | Se 0.60 (0.32,0.84)  Sp 0.97 (0.88,1.00)  PPV 0.82  NPV 0.91  LR+ 18  LR- 0.414 | n.a. |
| Filippou 2014(80) | 42 patients | Knee | Cohort study | MA | Consecutive patients waiting to undergo knee replacement surgery | US Diagnosis of CPPD | Se 0.96 (0.80,1.00)  Sp 0.88 (0.62,0.98)  PPV 0,92  NPV 0,93  LR+ 7,692  LR- 0.044 | n.a. |
| Juge 2014(81) | 16 Cases  16 Controls | Knee | Case control | SFA | Patients with painful knee effusion. The final diagnosis of CPPD was  proven by identification of CPP crystals in SFA. | US Diagnosis of CPPD | Se 1.00 (0.79,1.00)  Sp 0.88 (0.62,0.98)  PPV 0.89 (0.69,0.97)  NPV 1.00 | 0.87 |
| Contant 2014(87) | 66 patients | The single involved joint | Cohort Study | SFA | Consecutive patients with o mono-oligo or polyarthritis of unknown origin and with available joint fluid for analysis | US Diagnosis of CPPD | Se 0.73  Sp 0.58  PPV 0.59  NPV 0.72 | n.a. |
| Contant 2014(87) | 66 patients | The single involved joint | Cohort Study | Clinical Evaluation | Consecutive patients with o mono-oligo or polyarthritis of unknown origin and with available joint fluid for analysis | US Diagnosis of CPPD | Se 0.86  Sp 0.66  PPV 0.65  NPV 0.86 | n.a. |
| Contant 2014(87) | 66 patients | All joints (not only the one affected) | Cohort Study | SFA | Consecutive patients with o mono-oligo or polyarthritis of unknown origin and with available joint fluid for analysis | US Diagnosis of CPPD | Se 0.93  Sp 0.39  PPV 0.56  NPV 0.87 | n.a. |
| Contant 2014(87) | 66 patients | All joints (not only the one affected) | Cohort Study | Clinical Evaluation | Consecutive patients with o mono-oligo or polyarthritis of unknown origin and with available joint fluid for analysis | US Diagnosis of CPPD | Se 1.00  Sp 41  PPV 0.54  NPV 1.00 | n.a. |
| Ottaviani 2015(82) | 51 patients | Knee | Cohort study | SFA | Consecutive patients with knee effusion, divided in two groups according to the SFA analysis (CPPD+ vs CPPD-) | US Diagnosis of CPPD | Se: 1.00 (0.86,1.00)  Sp 0.92 (0.75,0.99)  PPV: 0.92 (0.77,0.98)  NPV: 1  LR+ 12.99  LR- 0.077 | 0.87 |
| Salcion 2015(88) | 78 patients | Knee | Cohort study | SFA | Consecutive patients affected by Knee OA and with synovial effusion requiring an arthrocentesis | US Diagnosis of CPPD | Se 0.95 (0.77,1.00)  Sp 0.66 (0.52,0.78)  PPV 0.52 (0.43,0.62)  NPV 0.97 (0.84,1.00) | n.a. |
| Forien 2017(84) | 32 cases  26 controls | Wrists | Case Control | SFA | Two groups of patients: consecutive patients with CPP crystals at SFA and a second group of consecutive patients without CPP crystals | US Diagnosis of CPPD | Se 0.94 (0.79,0.99)  Sp:0.85 (0.65,0.96)  PPV 0.88 (0.75,0.95)  NPV 0.92 (0.74,0.98)  LR+ 6,1  LR- 0.07 | n.a. |
| Di Matteo 2017(83) | 36 Cases  48 Controls | Wrists | Case Control | Mc Carty criteria | Consecutive patients referred to the Outpatient Clinic: 36 patients affected by CPPD according to Mc Carty criteria, 48 controls affected by other rheumatological diseases | US Identification of CPP deposits | Se 0.92 (0.83,0.96)  Sp 0.81 (0.71,0.89)  PPV: 0.86 (0.80,0.91)  NPV: 0.84 (0.78,0.89)  LR+ 4,889  LR- 0.103 | n.a. |
| Adinolfi 2017(89) | 50 patients | knee | Cohort study | SFA | Consecutive patients with knee pain and joint effusion of any grade | US Diagnosis of CPPD | Se 0.86 (0.67,0.96)  Sp 0.91 (0.71,0.99)  PPV 0.92 (0.76,0.98)  NPV 0.83 (0.67,0.93)  LR+ 9,429  LR- 0.157 | n.a. |

***Gout***

***Flow-chart§***


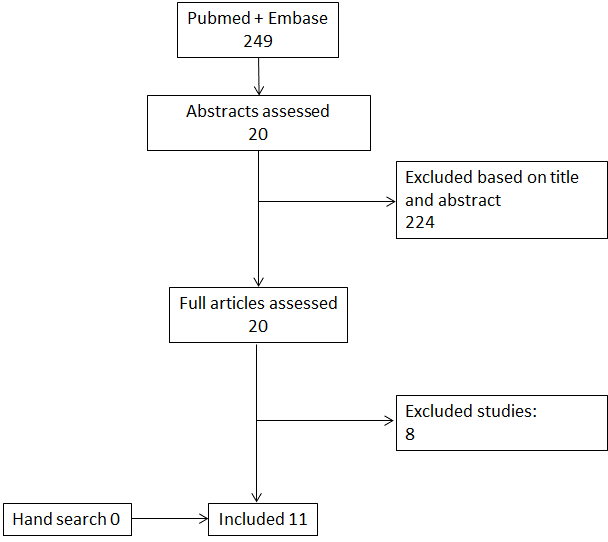


*§ 6 studies included from the previous review*

***Summary of findings table: US to diagnose gout elementary lesions***

Summary of findings of studies assessing the performance of US to diagnose gout lementary lesions. Estimates of diagnostic performance are presented as point estimate and 95% confidence intervals, unless specified. Se: sensitivity; Sp: specificity, LR+: positive likelihood ratio; LR-: negative likelihood ratio; MCP: metacarpophalangeal joints; PPV: positive predictive value; NPV: negative predictive value; MTP: metatarsophalangeal; DC: double contour; DCS: double contour sign; HCA: hyperechoic cloudy area; MSU: monosodium urate; HAG: hyperechoic aggregate.

| **Study** | **N** | **Site** | **Study design** | **Comparator/reference standard** | **Outcome** | | |
| --- | --- | --- | --- | --- | --- | --- | --- |
|  |  |  |  |  |  | **Diagnostic performance** | **Reliability (kappa)** |
| De Miguel E 2012 (90) | 26 | Knee, I MTP | Cross-sectional | Synovial fluid analysis | DCS or HCA | Se 1.0 (0.84, 1.0)  Sp 0.88 (0.67, 0.96)  PPV 0.82, NPV 1.0  LR-5.5 | 0.79-0.93 |
| Rettenbacher T 2008 (91) | 105 | Symptomatic joint/tendon | Longitudinal | X-ray | HCA  Bright foci  Hypervascularization  Erosion | Se 0.96 (0.90, 0.99)  Sp 0.73 (0.60, 0.84)  PPV 0.86, NPV 0.91 | n.a. |
| Lamers-Karnebeek FBG 2014 (92) | 54 | Symptomatic joint and the contra lateral side, 1^st^ MTP, kness | Cross-sectional | Synovial fluid analysis  (presence of MSU crystals) | DC  Snow storm  Tophi  All the three features | Se 0.77 (0.72, 0.81)  Sp 0.75 (0.66, 0.84)  PPV 0.74 (0.70, 0.79)  NPV 0.78 (0.74, 0.82)  LR+ 3.08, LR-0.31  Se 0.38 (0.34, 0.42)  Sp 0.86 (0.83, 0.89)  PPV 0.71 (0.66, 0.76)  NPV 0.60 (0.57, 0.63)  LR+ 2.6, LR-0.72  Se 0.19 (0.17, 0.22)  Sp 0.93 (0.91, 0.95)  PPV 0.71 (0.66, 0.76)  NPV 0.55 (0.49, 0.61)  LR+ 2.69, LR-0.87  Se 0.96 (0.95, 0.97)  Sp 0.68 (0.63, 0.73)  PPV 0.74 (0.70, 0.79)  NPV 0.95 (0.94, 0.96)  LR+ 2.99, LR-0.06 | Inter-reader 0.62 |
| Naredo E 2014 (93) | 133 | 1 joint and two tendons for HAG, 3 articular cartilage for DC | Longitudinal | Synovial fluid analysis  (presence of MSU crystals) | HAG or DC | Se 84.6  Sp 83.3 | Intra-reader 0.75  Inter-reader 0.52 |
| Pattamapaspong N 2017(94) | 89 | Symptomatic joint | Longitudinal | Synovial fluid analysis (presence of MSU crystals) | DC  HAG  Tophi  All the three features | Se 0.41 (0.31, 0.52)  Sp 0.91 (0.83, 0.96)  Se 0.58 (0.47, 0.68)  Sp 0.92 (0.83, 0,96)  Se 0.39 (0.29, 0.51)  Sp 1.0 (0.95, 1.0)  Se 0.75 (0.65, 0.84)  Sp 0.89 (0.80, 0.94)  PPV 0.91, NPV 0.71 | Inter-reader DC 0.63  Inter-reader HAG 0.58  Intra-reader tophi 0.74 |
| Das S 2017(95) | 92 | 1^st^ MTP and knees  (intercritic phase) | Cross-sectional | Synovial fluid analysis (presence of MSU crystals) | DC  Tophi | Se 0.69 (0.56, 0.80)  Sp 1.0 (0.88, 1.0)  Se 0.66 (0.55, 0.75)  Sp 1.0 (0.95, 1.0) | Intra-reader DC 1.0 (p 0.002)  Intra-reader tophi 0.74 (p < 0.001) |

***Summary of findings table: US to diagnose gout***

Summary of findings of studies assessing the performance of US to diagnose OA. Estimates of diagnostic performance are presented as point estimate and 95% confidence intervals, unless specified. Se: sensitivity; Sp: specificity; PPV: positive predictive value; NPV: negative predictive value; MSU: monosodium urate.

| **Study** | **N** | **Site** | **Study design** | **Comparator/reference standard** | **Population** | **Outcome** | | |
| --- | --- | --- | --- | --- | --- | --- | --- | --- |
|  |  |  |  |  |  |  | **Diagnostic performance** | **Reliability (kappa)** |
| Lai KL 2011(96) | 80 | Symptomatic joint | Retrospective | Synovial fluid analysis  (presence of MSU crystals) | Consecutive patients with mono-oligo arthritis | Intracellular MSU crystals | Se 0.23  Sp 1.0 | 0.73-0.86 |
| Huppertz A 2014(97) | 60 | Feet, knees, hand and elbow | Prospective | Synovial fluid analysis (presence of MSU crystals) | Consecutive patients referred for a clinical suspicion of gout | MSU crystals | Se 1.0 (0,93, 1.0)  Sp 0.8 (0.76, 0.94) | n.a. |
| Elsaman A 2016(98) | 100 | Knee, 1^st^ MTP | Cross-sectional | Synovial fluid analysis  (presence of MSU crystals) | Consecutive patients with mono-oligo arthritis | MSU crystals | Se 0.86 (0.78, 0.91)  Sp 0.87 (0.89, 0.92) | n.a. |
| Gruber M 2013(99) | 21 | Hands, wrists, elbows, ankle and feet | Cross-sectional cohort | Synovial fluid analysis  (presence of MSU crystals) | Consecutive patients referred for a clinical suspicion of gout (acute or chronic) | Clinical diagnosis or MSU crystals (when available) | Se 0.42 (0.26, 0.59) | n.a. |
| Pascal Z 2015 (100) | 109 | Knee, 1^st^ MTP, ankles | Cross-sectional | Synovial fluid analysis (presence of MSU crystals) | Consecutive patients referred for a clinical suspicion of microcrystalline arthritis | MSU crystals | Se 0.60 (single symptomatic site)  Se 0.84 (multiple sites)  Sp 0.92 (single symptomatic site)  0.78 (multiple sites) | n.a. |

**References**

1. Iagnocco A., Filippucci E., Ossandon A., Ciapetti A., Salaffi F., Basili S., et al. High resolution ultrasonography in detection of bone erosions in patients with hand osteoarthritis. J Rheumatol. 2005;32(12):2381–3.

2. Keen H.I., Lavie F., Wakefield R.J., D’Agostino M.-A., Berner Hammer H., Hensor E.M.A., et al. The development of a preliminary ultrasonographic scoring system for features of hand osteoarthritis. Ann Rheum Dis. 2008;67(5):651–5.

3. Koutroumpas A.C., Alexiou I.S., Vlychou M., Sakkas L.I. Comparison between clinical and ultrasonographic assessment in patients with erosive osteoarthritis of the hands. Clin Rheumatol. 2010;29(5):511–6.

4. Mathiessen A., Haugen I.K., Slatkowsky-Christensen B., Bøyesen P., Kvien T.K., Hammer H.B. Ultrasonographic assessment of osteophytes in 127 patients with hand osteoarthritis: Exploring reliability and associations with MRI, radiographs and clinical joint findings. Ann Rheum Dis. 2013;72(1):51–6.

5. Vlychou M., Koutroumpas A., Malizos K., Sakkas L.I. Ultrasonographic evidence of inflammation is frequent in hands of patients with erosive osteoarthritis. Osteoarthritis Cartilage. 2009;17(10):1283–7.

6. Vlychou M, Koutroumpas A, Alexiou I, Fezoulidis I, Sakkas LI. High-resolution ultrasonography and 3.0 T magnetic resonance imaging in erosive and nodal hand osteoarthritis: high frequency of erosions in nodal osteoarthritis. Clin Rheumatol. 2013 Jun;32(6):755–62.

7. Wittoek R, Carron P, Verbruggen G. Structural and inflammatory sonographic findings in erosive and non-erosive osteoarthritis of the interphalangeal finger joints. Ann Rheum Dis. 2010 Dec;69(12):2173–6.

8. Wittoek R., Jans L., Lambrecht V., Carron P., Verstraete K., Verbruggen G. Reliability and construct validity of ultrasonography of soft tissue and destructive changes in erosive osteoarthritis of the interphalangeal finger joints: A comparison with MRI. Ann Rheum Dis. 2011;70(2):278–83.

9. Iagnocco A, Coari G. Usefulness of high resolution US in the evaluation of effusion in osteoarthritic first carpometacarpal joint. Scand J Rheumatol. 2000;29(3):170–3.

10. Akgul O., Guldeste Z., Ozgocmen S. The reliability of the clinical examination for detecting Baker’s cyst in asymptomatic fossa. Int J Rheum Dis. 2014;17(2):204–9.

11. Chatzopoulos D, Moralidis E, Markou P, Makris V, Arsos G. Baker’s cysts in knees with chronic osteoarthritic pain: a clinical, ultrasonographic, radiographic and scintigraphic evaluation. Rheumatol Int. 2008 Dec;29(2):141–6.

12. Eşen S, Akarırmak U, Aydın FY, Unalan H. Clinical evaluation during the acute exacerbation of knee osteoarthritis: the impact of diagnostic ultrasonography. Rheumatol Int. 2013 Mar;33(3):711–7.

13. Ike R.W., Somers E.C., Arnold E.L., Arnold W.J. Ultrasound of the knee during voluntary quadriceps contraction: A technique for detecting otherwise occult effusions. Arthritis Care Res. 2010;62(5):725–9.

14. Cl L, Mh H, Cy C, Ch C, Jy S, Yc T. The Validity of in Vivo Ultrasonographic Grading of Osteoarthritic Femoral Condylar Cartilage: A Comparison With in Vitro Ultrasonographic and Histologic Gradings [Internet]. Osteoarthritis and cartilage. 2008 [cited 2019 Dec 17]. Available from: https://pubmed.ncbi.nlm.nih.gov/17920940/?from_term=The+validity+of+in+vivo+ultrasonographic+grading+of+osteoarthritic+femoral+condylar+cartilage%3A+a+comparison+with+in+vitro+ultrasonographic+and+histologic+gradings&from_pos=1

15. Pendleton A, Millar A, O’Kane D, Wright GD, Taggart AJ. Can sonography be used to predict the response to intra-articular corticosteroid injection in primary osteoarthritis of the knee? Scand J Rheumatol. 2008 Oct;37(5):395–7.

16. Song I.H., Burmester G.R., Backhaus M., Althoff C.E., Hermann K.G., Scheel A.K., et al. Knee osteoarthritis. Efficacy of a new method of contrast-enhanced musculoskeletal ultrasonography in detection of synovitis in patients with knee osteoarthritis in comparison with magnetic resonance imaging. Ann Rheum Dis. 2008;67(1):19–25.

17. Yoon C-H, Kim H-S, Ju JH, Jee W-H, Park S-H, Kim H-Y. Validity of the sonographic longitudinal sagittal image for assessment of the cartilage thickness in the knee osteoarthritis. Clin Rheumatol. 2008 Dec;27(12):1507–16.

18. Okano T., Filippucci E., Carlo M.D., Draghessi A., Carotti M., Salaffi F., et al. Ultrasonographic evaluation of joint damage in knee osteoarthritis: Feature-specific comparisons with conventional radiography. Rheumatol U K. 2016;55(11):2040–9.

19. Camerer M., Ehrenstein B., Hoffstetter P., Fleck M., Hartung W. High-resolution ultrasound of the midfoot: sonography is more sensitive than conventional radiography in detection of osteophytes and erosions in inflammatory and non-inflammatory joint disease. Clin Rheumatol. 2017;36(9):2145–9.

20. Mortada M., Zeid A., Abd El-Hamid Al-Toukhy M., Ezzeldin N., Elgawish M. Reliability of a proposed ultrasonographic grading scale for severity of primary knee osteoarthritis. Clin Med Insights Arthritis Musculoskelet Disord. 2016;9((Mortada M., m_a_mortada@yahoo.com; Abd El-Hamid Al-Toukhy M.; Ezzeldin N.; Elgawish M.) Rheumatology and Rehabilitation Department, Faculty of Medicine, Zagazig University, Zagazig, Egypt):161–6.

21. Matsos M., Harish S., Zia P., Ho Y., Chow A., Ioannidis G., et al. Ultrasound of the hands and feet for rheumatological disorders: Influence on clinical diagnostic confidence and patient management. Skeletal Radiol. 2009;38(11):1049–54.

22. Zayat A.S., Ellegaard K., Conaghan P.G., Terslev L., Hensor E.M.A., Freeston J.E., et al. The specificity of ultrasound-detected bone erosions for rheumatoid arthritis. Ann Rheum Dis. 2015;74(5):897–903.

23. Salaffi F., Ciapetti A., Gasparini S., Carotti M., Filippucci E., Grassi W. A clinical prediction rule combining routine assessment and power doppler ultrasonography for predicting progression to rheumatoid arthritis from early-onset undifferentiated arthritis. Clin Exp Rheumatol. 2010;28(5):686–94.

24. Filer A., De Pablo P., Allen G., Nightingale P., Jordan A., Jobanputra P., et al. Utility of ultrasound joint counts in the prediction of rheumatoid arthritis in patients with very early synovitis. Ann Rheum Dis. 2011;70(3):500–7.

25. Tămaş M-M, Rednic N, Felea I, Rednic S. Ultrasound assessment for the rapid classification of early arthritis patients. J Investig Med Off Publ Am Fed Clin Res. 2013 Dec;61(8):1184–91.

26. Navalho M., Resende C., Rodrigues A.M., Alberto Pereira Da Silva J., Fonseca J.E., Campos J., et al. Bilateral evaluation of the hand and wrist in untreated early inflammatory arthritis: A comparative study of ultrasonography and magnetic resonance imaging. J Rheumatol. 2013;40(8):1282–92.

27. Kawashiri S.-Y., Suzuki T., Okada A., Yamasaki S., Tamai M., Nakamura H., et al. Musculoskeletal ultrasonography assists the diagnostic performance of the 2010 classification criteria for rheumatoid arthritis. Mod Rheumatol. 2013;23(1):36–43.

28. Ozgul A., Yasar E., Arslan N., Balaban B., Taskaynatan M.A., Tezel K., et al. The comparison of ultrasonographic and scintigraphic findings of early arthritis in revealing rheumatoid arthritis according to criteria of American College of Rheumatology. Rheumatol Int. 2009;29(7):765–8.

29. Nakagomi D., Ikeda K., Okubo A., Iwamoto T., Sanayama Y., Takahashi K., et al. Ultrasound can improve the accuracy of the 2010 American College of Rheumatology/European League against rheumatism classification criteria for rheumatoid arthritis to predict the requirement for methotrexate treatment. Arthritis Rheum. 2013;65(4):890–8.

30. Broll M., Albrecht K., Tarner I., Müller-Ladner U., Strunk J. Sensitivity and specificity of ultrasonography and low-field magnetic resonance imaging for diagnosing arthritis. Clin Exp Rheumatol. 2012;30(4):543–7.

31. Platt P.N., Pratt A. The predictive value of musculoskeletal ultrasound in unselected early arthritis clinic patients with polyarthralgia. Rheumatol U K. 2013;52((Platt P.N.; Pratt A.) Rheumatology, Freeman Hospital, Newcastle, United Kingdom):i65.

32. Rezaei H, Torp-Pedersen S, af Klint E, Backheden M, Kisten Y, Györi N, et al. Diagnostic utility of musculoskeletal ultrasound in patients with suspected arthritis--a probabilistic approach. Arthritis Res Ther. 2014 Oct 1;16(5):448.

33. Minowa K, Ogasawara M, Murayama G, Gorai M, Yamada Y, Nemoto T, et al. Predictive grade of ultrasound synovitis for diagnosing rheumatoid arthritis in clinical practice and the possible difference between patients with and without seropositivity. Mod Rheumatol. 2015 Jul 3;1–6.

34. Zufferey P, Rebell C, Benaim C, Ziswiler HR, Dumusc A, So A. Ultrasound can be useful to predict an evolution towards rheumatoid arthritis in patients with inflammatory polyarthralgia without anticitrullinated antibodies. Joint Bone Spine. 2017 May;84(3):299–303.

35. Ji L., Deng X., Geng Y., Song Z., Zhang Z. The additional benefit of ultrasonography to 2010 ACR/EULAR classification criteria when diagnosing rheumatoid arthritis in the absence of anti-cyclic citrullinated peptide antibodies. Clin Rheumatol. 2017;36(2):261–7.

36. Freeston J.E., Coates L.C., Helliwell P.S., Hensor E.M.A., Wakefield R.J., Emery P., et al. Is there subclinical enthesitis in early psoriatic arthritis? a clinical comparison with power doppler ultrasound. Arthritis Care Res. 2012;64(10):1617–21.

37. Gutierrez M, Filippucci E, Salaffi F, Di Geso L, Grassi W. Differential diagnosis between rheumatoid arthritis and psoriatic arthritis: the value of ultrasound findings at metacarpophalangeal joints level. Ann Rheum Dis. 2011 Jun;70(6):1111–4.

38. Iagnocco A, Spadaro A, Marchesoni A, Cauli A, De Lucia O, Gabba A, et al. Power Doppler ultrasonographic evaluation of enthesitis in psoriatic arthritis. A multi-center study. Joint Bone Spine. 2012 May;79(3):324–5.

39. Lin Z, Wang Y, Mei Y, Zhao Y, Zhang Z. High-Frequency Ultrasound in the Evaluation of Psoriatic Arthritis: A Clinical Study. Am J Med Sci. 2015 Jul;350(1):42–6.

40. Marchesoni A., De Lucia O., Rotunno L., De Marco G., Manara M. Entheseal power Doppler ultrasonography: A comparison of psoriatic arthritis and fibromyalgia. J Rheumatol. 2012;39(SUPPL. 89):29–31.

41. Melchiorre D., Calderazzi A., Maddali Bongi S., Cristofani R., Bazzichi L., Eligi C., et al. A comparison of ultrasonography and magnetic resonance imaging in the evaluation of temporomandibular joint involvement in rheumatoid arthritis and psoriatic arthritis. Rheumatology. 2003;42(5):673–6.

42. Turner D.E., Hyslop E., Barn R., Mcinnes I.B., Steultjens M.P.M., Woodburn J. Metatarsophalangeal joint pain in psoriatic arthritis: A cross-sectional study. Rheumatol U K. 2014;53(4):737–40.

43. Wiell C., Szkudlarek M., Hasselquist M., Møller J.M., Vestergaard A., Nørregaard J., et al. Ultrasonography, magnetic resonance imaging, radiography, and clinical assessment of inflammatory and destructive changes in fingers and toes of patients with psoriatic arthritis. Arthritis Res Ther [Internet]. 2007;9(6). Available from: http://www.embase.com/search/results?subaction=viewrecord&from=export&id=L350263515

44. Woodburn J, Hyslop E, Barn R, McInnes IB, Turner DE. Achilles tendon biomechanics in psoriatic arthritis patients with ultrasound proven enthesitis. Scand J Rheumatol. 2013;42(4).

45. Acquacalda E., Albert C., Montaudie H., Fontas E., Danre A., Roux C.H., et al. Ultrasound study of entheses in psoriasis patients with or without musculoskeletal symptoms: A prospective study. Joint Bone Spine. 2015;82(4):267–71.

46. Eder L., Jayakar J., Thavaneswaran A., Haddad A., Chandran V., Salonen D., et al. Is the madrid sonographic enthesitis index useful for differentiating psoriatic arthritis from psoriasis alone and healthy controls? J Rheumatol. 2014;41(3):466–72.

47. Ciancio G, Volpinari S, Fotinidi M, Furini F, Farina I, Bortoluzzi A, et al. Involvement of the inconstant bursa of the fifth metatarsophalangeal joint in psoriatic arthritis: a clinical and ultrasonographic study. BioMed Res Int. 2014;2014.

48. Falcao S., de Miguel E., Castillo-Gallego C., Peiteado D., Branco J., Martín Mola E. Achilles enthesis ultrasound: The importance of the bursa in spondyloarthritis. Clin Exp Rheumatol. 2013;31(3):422–7.

49. Ezzat Y, Gaber W, Abd ELRSF, Ezzat M, El Sayed M. Ultrasonographic evaluation of lower limb enthesis in patients with early spondyloarthropathies. Egypt Rheumatol. 2013;35(1):29–35.

50. Bandinelli F, Prignano F, Bonciani D, Bartoli F, Collaku L, Candelieri A, et al. Ultrasound detects occult entheseal involvement in early psoriatic arthritis independently of clinical features and psoriasis severity. Clin Exp Rheumatol. 2013 Apr;31(2):219–24.

51. Aydin SZ, Castillo-Gallego C, Ash ZR, Abignano G, Marzo-Ortega H, Wittmann M, et al. Potential use of optical coherence tomography and high-frequency ultrasound for the assessment of nail disease in psoriasis and psoriatic arthritis. Dermatol Basel Switz. 2013;227(1).

52. Ash Z., Hodgson R., Grainger A., Aydin S.Z., Castillo-Gallego C., Tan A.L., et al. Imaging of psoriatic nail disease pre and post anti-TNF therapy shows persistent subclinical inflammation despite good clinical response. BMC Musculoskelet Disord [Internet]. 2013;14((Ash Z., Z.Ash@leeds.ac.uk; Hodgson R.; Grainger A.; Aydin S.Z.; Castillo-Gallego C.; Tan A.L.; Marzo-Ortega H.; McGonagle D.) Leeds Institute of Molecular Medicine, University of Leeds, Leeds Teaching Hospitals, Leeds, United Kingdom). Available from: http://www.embase.com/search/results?subaction=viewrecord&from=export&id=L71270647

53. Aydin S.Z., Castillo-Gallego C., Ash Z.R., Marzo-Ortega H., Emery P., Wakefield R.J., et al. Ultrasonographic assessment of nail in psoriatic disease shows a link between onychopathy and distal interphalangeal joint extensor tendon enthesopathy. Dermatology. 2012;225(3):231–5.

54. De Simone C, Caldarola G, D’Agostino M, Carbone A, Guerriero C, Bonomo L, et al. Usefulness of ultrasound imaging in detecting psoriatic arthritis of fingers and toes in patients with psoriasis. Clin Dev Immunol. 2011;2011.

55. Farouk H.M., Mostafa A.A.A., Youssef S.S., Elbeblawy M.M.S., Assaf N.Y., Elokda E.S.E. Value of entheseal ultrasonography and serum cartilage oligomeric matrix protein in the preclinical diagnosis of psoriatic arthritis. Clin Med Insights Arthritis Musculoskelet Disord. 2010;3((Farouk H.M., dr_hananfarouk@hotmail.com) Internal Medicine and Rheumatology, Faculty of Medicine Ain Shams University, Cairo, Egypt):7–14.

56. Falsetti P., Frediani B., Fioravanti A., Acciai C., Baldi F., Filippou G., et al. Sonographic study of calcaneal entheses in erosive osteoarthritis, nodal osteoarthritis, rheumatoid arthritis and psoriatic arthritis. Scand J Rheumatol. 2003;32(4):229–34.

57. Falsetti P, Frediani B, Filippou G, Acciai C, Baldi F, Storri L, et al. Enthesitis of proximal insertion of the deltoid in the course of seronegative spondyloarthritis. An atypical enthesitis that can mime impingement syndrome. Scand J Rheumatol. 2002;31(3):158–62.

58. Fournie B., Margarit-Coll N., Champetier de Ribes T.L., Zabraniecki L., Jouan A., Vincent V., et al. Extrasynovial ultrasound abnormalities in the psoriatic finger. Prospective comparative power-doppler study versus rheumatoid arthritis. Joint Bone Spine. 2006;73(5):527–31.

59. Mendonça JA. [Differences of spectral Doppler in psoriatic arthritis and onychomycosis]. Rev Bras Reumatol. 2014 Dec;54(6):490–3.

60. Zabotti A, Salvin S, Quartuccio L, De Vita S. Differentiation between early rheumatoid and early psoriatic arthritis by the ultrasonographic study of the synovio-entheseal complex of the small joints of the hands. Clin Exp Rheumatol. 2016 Jun;34(3):459–65.

61. Groves C, Chandramohan M, Chew NS, Aslam T, Helliwell PS. Clinical Examination, Ultrasound and MRI Imaging of The Painful Elbow in Psoriatic Arthritis and Rheumatoid Arthritis: Which is Better, Ultrasound or MR, for Imaging Enthesitis? Rheumatol Ther. 2017 Jun;4(1):71–84.

62. Dasgupta B, Cimmino MA, Maradit-Kremers H, Schmidt WA, Schirmer M, Salvarani C, et al. 2012 provisional classification criteria for polymyalgia rheumatica: a European League Against Rheumatism/American College of Rheumatology collaborative initiative. Ann Rheum Dis. 2012 Apr;71(4):484–92.

63. Ruta S, Rosa J, Navarta DA, Saucedo C, Catoggio LJ, Monaco RG, et al. Ultrasound assessment of new onset bilateral painful shoulder in patients with polymyalgia rheumatica and rheumatoid arthritis. Clin Rheumatol. 2012 Sep;31(9):1383–7.

64. P F, C A, A V, L L. Ultrasonography in Early Assessment of Elderly Patients With Polymyalgic Symptoms: A Role in Predicting Diagnostic Outcome? [Internet]. Scandinavian journal of rheumatology. 2011 [cited 2019 Dec 17]. Available from: https://pubmed.ncbi.nlm.nih.gov/20653466/?from_term=fALSETTI+P+2011&from_size=10&from_pos=1

65. Cantini F, Salvarani C, Olivieri I, Niccoli L, Padula A, Macchioni L, et al. Shoulder ultrasonography in the diagnosis of polymyalgia rheumatica: a case-control study. J Rheumatol. 2001 May;28(5):1049–55.

66. Frediani B, Falsetti P, Storri L, Bisogno S, Baldi F, Campanella V, et al. Evidence for synovitis in active polymyalgia rheumatica: sonographic study in a large series of patients. J Rheumatol. 2002 Jan;29(1):123–30.

67. Cantini F, Niccoli L, Nannini C, Padula A, Olivieri I, Boiardi L, et al. Inflammatory changes of hip synovial structures in polymyalgia rheumatica. Clin Exp Rheumatol. 2005 Aug;23(4):462–8.

68. Coari G, Paoletti F, Iagnocco A. Shoulder involvement in rheumatic diseases. Sonographic findings. J Rheumatol. 1999 Mar;26(3):668–73.

69. Lange U, Piegsa M, Teichmann J, Neeck G. Ultrasonography of the glenohumeral joints--a helpful instrument in differentiation in elderly onset rheumatoid arthritis and polymyalgia rheumatica. Rheumatol Int. 2000;19(5):185–9.

70. Macchioni P., Boiardi L., Catanoso M., Pazzola G., Salvarani C. Performance of the new 2012 EULAR/ACR classification criteria for polymyalgia rheumatica: Comparison with the previous criteria in a single-centre study. Ann Rheum Dis. 2014;73(6):1190–3.

71. Weigand S., Ehrenstein B., Fleck M., Hartung W. Joint involvement in patients with early polymyalgia rheumatica using high-resolution ultrasound and its contribution to the EULAR/ACR 2012 classification criteria for polymyalgia rheumatica. J Rheumatol. 2014;41(4):730–4.

72. Coari G, Iagnocco A, Zoppini A. Chondrocalcinosis: sonographic study of the knee. Clin Rheumatol. 1995 Sep;14(5):511–4.

73. Foldes K. Knee chondrocalcinosis: An ultrasonographic study of the hyalin cartilage. Clin Imaging. 2002;26(3):194–6.

74. Falsetti P, Frediani B, Acciai C, Baldi F, Filippou G, Prada EP, et al. Ultrasonographic study of Achilles tendon and plantar fascia in chondrocalcinosis. J Rheumatol. 2004 Nov;31(11):2242–50.

75. Filippucci E., Gutierrez Riveros M., Georgescu D., Salaffi F., Grassi W. Hyaline cartilage involvement in patients with gout and calcium pyrophosphate deposition disease. An ultrasound study. Osteoarthritis Cartilage. 2009;17(2):178–81.

76. Ellabban AS, Kamel SR, Abo Omar HAS, El-Sherif AMH, Abdel-Magied RA. Ultrasonographic findings of Achilles tendon and plantar fascia in patients with calcium pyrophosphate deposition disease. Clin Rheumatol. 2012 Apr;31(4):697–704.

77. Filippou G., Bozios P., Gambera D., Lorenzini S., Bertoldi I., Adinolfi A., et al. Ultrasound detection of calcium pyrophosphate dihydrate crystal deposits in menisci: A pilot in vivo and ex vivo study. Ann Rheum Dis. 2012;71(8):1426–7.

78. Barskova VG, Kudaeva FM, Bozhieva LA, Smirnov AV, Volkov AV, Nasonov EL. Comparison of three imaging techniques in diagnosis of chondrocalcinosis of the knees in calcium pyrophosphate deposition disease. Rheumatol Oxf Engl. 2013 Jun;52(6):1090–4.

79. Gutierrez M., Di Geso L., Salaffi F., Carotti M., Girolimetti R., De Angelis R., et al. Ultrasound detection of cartilage calcification at knee level in calcium pyrophosphate deposition disease. Arthritis Care Res. 2014;66(1):69–73.

80. Filippou G., Adinolfi A., Lorenzini S., Bertoldi I., Di Sabatino V., Picerno V., et al. Ultrasound versus X-rays versus synovial fluid analysis for the diagnosis of calcium pyrophosphate dihydrate deposition disease: Is it CPPD? Arthritis Rheumatol. 2014;66((Filippou G.; Adinolfi A.; Bertoldi I.; Di Sabatino V.; Picerno V.; Galeazzi M.; Frediani B.) University of Siena, Siena, Italy):S77–8.

81. Juge P.-A., Ottaviani S., Aubrun A., Palazzo E., Dieudé P. Sensitivity and reproducibility of ultrasonography in calcium pyrophosphate crystal deposition: A case-control study. Ann Rheum Dis [Internet]. 2014;73((Juge P.-A.; Ottaviani S.; Aubrun A.; Palazzo E.; Dieudé P.) Rheumatology, Hôpital Bichat, Paris, France). Available from: http://www.embase.com/search/results?subaction=viewrecord&from=export&id=L71552755

82. Ottaviani S., Juge P.-A., Aubrun A., Palazzo E., Dieudé P. Sensitivity and reproducibility of ultrasonography in calcium pyrophosphate crystal deposition in knee cartilage: A cross-sectional study. J Rheumatol. 2015;42(8):1511–3.

83. Di Matteo A., Filippucci E., Salaffi F., Carotti M., Carboni D., Di Donato E., et al. Diagnostic accuracy of musculoskeletal ultrasound and conventional radiography in the assessment of the wrist triangular fibrocartilage complex in patients with definite diagnosis of calcium pyrophosphate dihydrate deposition disease. Clin Exp Rheumatol. 2017;35(4):647–52.

84. Forien M., Combier A., Gardette A., Palazzo E., Dieudé P., Ottaviani S. Comparison of ultrasonography and radiography of the wrist for diagnosis of calcium pyrophosphate deposition. Joint Bone Spine [Internet]. 2017;((Forien M.; Combier A.; Gardette A.; Palazzo E.; Dieudé P.; Ottaviani S., sebastien.ottaviani@aphp.fr) Service de rhumatologie, hôpital Bichat, université Paris Diderot, AP-HP, 46, rue Henri-Huchard, 75018 Paris, France). Available from: http://www.embase.com/search/results?subaction=viewrecord&from=export&id=L619456834

85. Filippou G, Frediani B, Gallo A, Menza L, Falsetti P, Baldi F, et al. A ‘new’ technique for the diagnosis of chondrocalcinosis of the knee: sensitivity and specificity of high-frequency ultrasonography. Ann Rheum Dis. 2007 Aug;66(8):1126–8.

86. Ruta S., Catay E., Marin J., Rosa J., García-Monaco R., Soriano E.R. Knee effusion: Ultrasound as a useful tool for the detection of calcium pyrophosphate crystals. Clin Rheumatol. 2016;35(4):1087–91.

87. Contant E., Ornetti P., Bohm A., Fortunet C., Maillefert J.F. Interest of musculoskeletal ultrasound in the diagnosis of calcium pyrophosphate dihydrate deposition disease. Ann Rheum Dis [Internet]. 2014;73((Contant E.; Ornetti P.; Bohm A.; Fortunet C.; Maillefert J.F.) Rheumatology, University Hospital, Dijon, France). Available from: http://www.embase.com/search/results?subaction=viewrecord&from=export&id=L71552725

88. Salcion A., Kozyreff-Meurice M., Richette P., Avenel G., Bisson-Vaivre A., Trouvin A.-P., et al. Assessment of the ultrasonography’s efficiency as a diagnostic tool for calcium pyrophosphate crystal deposition disease. Ann Rheum Dis. 2015;74((Salcion A.) Rheumatology Department, Hôpital Cochin, Paris, France):538.

89. Adinolfi A, Picerno V, Scanu A, Toscano C, Scirè C, Carrara G, et al. Diagnostic performance of the new omeract criteria for cppd identification by us: correlation with synovial fluid analysis. 2017 Jun;730.2-731.

90. E DM, Jg P, C C, D P, Rj T, E M-M. Diagnosis of Gout in Patients With Asymptomatic Hyperuricaemia: A Pilot Ultrasound Study [Internet]. Annals of the rheumatic diseases. 2012 [cited 2019 Dec 17]. Available from: https://pubmed.ncbi.nlm.nih.gov/21953340/?from_term=DE+MIGUEL+E+2011&from_pos=6

91. Rettenbacher T., Ennemoser S., Weirich H., Ulmer H., Hartig F., Klotz W., et al. Diagnostic imaging of gout: Comparison of high-resolution US versus conventional X-ray. Eur Radiol. 2008;18(3):621–30.

92. Lamers-Karnebeek F.B.G., Van Riel P.L.C.M., Jansen T.L. Additive value for ultrasonographic signal in a screening algorithm for patients presenting with acute mono-/oligoarthritis in whom gout is suspected. Clin Rheumatol. 2014;33(4):555–9.

93. E N, J U, M J-P, A M, E V, E B, et al. Ultrasound-detected Musculoskeletal Urate Crystal Deposition: Which Joints and What Findings Should Be Assessed for Diagnosing Gout? [Internet]. Annals of the rheumatic diseases. 2014 [cited 2019 Dec 17]. Available from: https://pubmed.ncbi.nlm.nih.gov/23709244/?from_single_result=naredo+e+2013+gout

94. Pattamapaspong N., Vuthiwong W., Kanthawang T., Louthrenoo W. Value of ultrasonography in the diagnosis of gout in patients presenting with acute arthritis. Skeletal Radiol. 2017;46(6):759–67.

95. Das S., Ghosh A., Ghosh P., Lahiri D., Sinhamahapatra P., Basu K. Sensitivity and specificity of ultrasonographic features of gout in intercritical and chronic phase. Int J Rheum Dis. 2017;20(7):887–93.

96. Lai K.-L., Chiu Y.-M. Role of Ultrasonography in Diagnosing Gouty Arthritis. J Med Ultrasound. 2011;19(1):7–13.

97. Huppertz A., Hermann K.-G.A., Diekhoff T., Wagner M., Hamm B., Schmidt W.A. Systemic staging for urate crystal deposits with dual-energy CT and ultrasound in patients with suspected gout. Rheumatol Int. 2014;34(6):763–71.

98. Elsaman A.M., Muhammad E.M.S., Pessler F. Sonographic Findings in Gouty Arthritis: Diagnostic Value and Association with Disease Duration. Ultrasound Med Biol. 2016;42(6):1330–6.

99. Gruber M, Bodner G, Rath E, Supp G, Weber M, Schueller-Weidekamm C. Dual-energy computed tomography compared with ultrasound in the diagnosis of gout. Rheumatol Oxf Engl. 2014 Jan;53(1):173–9.

100. Pascal Z., Valcov R., Fabreguet I., Dumusc A., Omoumi P., So A. A prospective evaluation of ultrasound as a diagnostic tool in acute microcrystalline arthritis. Arthritis Res Ther [Internet]. 2015;17(1). Available from: http://www.embase.com/search/results?subaction=viewrecord&from=export&id=L605235159
